# Short high fat diet triggers reversible and region specific effects in DCX^+^ hippocampal immature neurons of adolescent male mice

**DOI:** 10.1038/s41598-021-01059-y

**Published:** 2021-11-02

**Authors:** Fausto Chiazza, Heather Bondi, Irene Masante, Federico Ugazio, Valeria Bortolotto, Pier Luigi Canonico, Mariagrazia Grilli

**Affiliations:** 1 Laboratory of Neuroplasticity, University of Piemonte Orientale, Novara, Italy; 2 Department of Pharmaceutical Sciences, University of Piemonte Orientale, Novara, Italy

**Keywords:** Neurology, Neuroscience, Neurogenesis

## Abstract

Adolescence represents a crucial period for maturation of brain structures involved in cognition. Early in life unhealthy dietary patterns are associated with inferior cognitive outcomes at later ages; conversely, healthy diet is associated with better cognitive results. In this study we analyzed the effects of a short period of hypercaloric diet on newborn hippocampal doublecortin^+^ (DCX) immature neurons in adolescent mice. Male mice received high fat diet (HFD) or control low fat diet (LFD) from the 5th week of age for 1 or 2 weeks, or 1 week HFD followed by 1 week LFD. After diet supply, mice were either perfused for immunohistochemical (IHC) analysis or their hippocampi were dissected for biochemical assays. Detailed morphometric analysis was performed in DCX^+^ cells that displayed features of immature neurons. We report that 1 week-HFD was sufficient to dramatically reduce dendritic tree complexity of DCX^+^ cells. This effect occurred specifically in dorsal and not ventral hippocampus and correlated with reduced BDNF expression levels in dorsal hippocampus. Both structural and biochemical changes were reversed by a return to LFD. Altogether these studies increase our current knowledge on potential consequences of hypercaloric diet on brain and in particular on dorsal hippocampal neuroplasticity.

## Introduction

The consumption of hypercaloric and fat enriched diets is associated with an increased risk of metabolic disorders^1^, and is fueling a worldwide increase of these diseases^2^. In addition, unhealthy diets characterized by caloric consumption that far exceeds body energy expenditure have been associated with a major risk of developing neurodegenerative^3^ or psychiatric diseases^4^. Up to a third of all clinical cases of neurodegenerative disease are supposed to be ascribed to modifiable lifestyle risk factors, such as nutrition^5,6^.

A lifestyle characterized by detrimental nutritional behavior is particularly impacting at younger ages, during childhood and adolescence. Juvenile years are, indeed, a time of rapid cognitive development, but also of great vulnerability^7^. A systematic review of clinical literature showed that unhealthy dietary patterns early in life are associated with inferior cognitive outcomes at later ages. Conversely, a healthy diet is associated with better cognitive results^7^. Consumption of meals containing normo-caloric and fresh ingredients at young ages has been related with better cognitive performance during growth^8^. On the contrary, in adolescence a diet high in processed ingredients and added sugar has been correlated with lower school achievement, language and nonverbal reasoning^9,10^. Interestingly, several studies, both in human and preclinical models, also identified an association between food overconsumption and brain volume loss^11,12^.

In the central nervous system (CNS), memory and learning processes are regulated by multiple brain areas, but a predominant role is played by the hippocampus. This region is characterized by anatomical segregation of its functions along the dorso-ventral axis: dorsal hippocampus seems to be associated primarily with cognition, while ventral hippocampus relates to stress, emotion, and affect (reviewed in Ref.^13^). In the dentate gyrus (DG) of both dorsal and ventral hippocampus, postnatal hippocampal neurogenesis (hNG) takes place allowing newborn neurons to form and, with time, to integrate in the hippocampal circuitry and contribute to its functionality^14^. A large body of data suggests that, when dysregulated, hNG contributes to cognitive impairment and mood alterations^15,16^. Chronic overnutrition is known to have a negative effect on this form of neuroplasticity, causing a reduction in the number of proliferating cells and of newborn adult hippocampal neurons^17^. An important step of hNG is the formation of cells expressing doublecortin (DCX), a protein essential for neuronal differentiation and migration^18^. Recent experimental evidence in murine models showed that the number of DCX^+^ cells is reduced by chronic periods of overnutrition^19,20^, and these deleterious effects have a higher magnitude at younger ages^21^, and may be region-specific^22^. Moreover, some of these alterations occur before a significant weight gain^24–28^. These findings suggest that overnutrition may directly affect brain structures, independently of metabolic derangements. Based on these observations and in light of the potential relevance in pathophysiology, herein we investigated the effects of a short period of a HFD on the fine structural architecture of hippocampal DCX^+^ immature neurons in adolescent mice.

## Results

### Seven days of HFD affects cellular complexity of DCX immunolabeled cells in dorsal hippocampus

**Figure 1:**
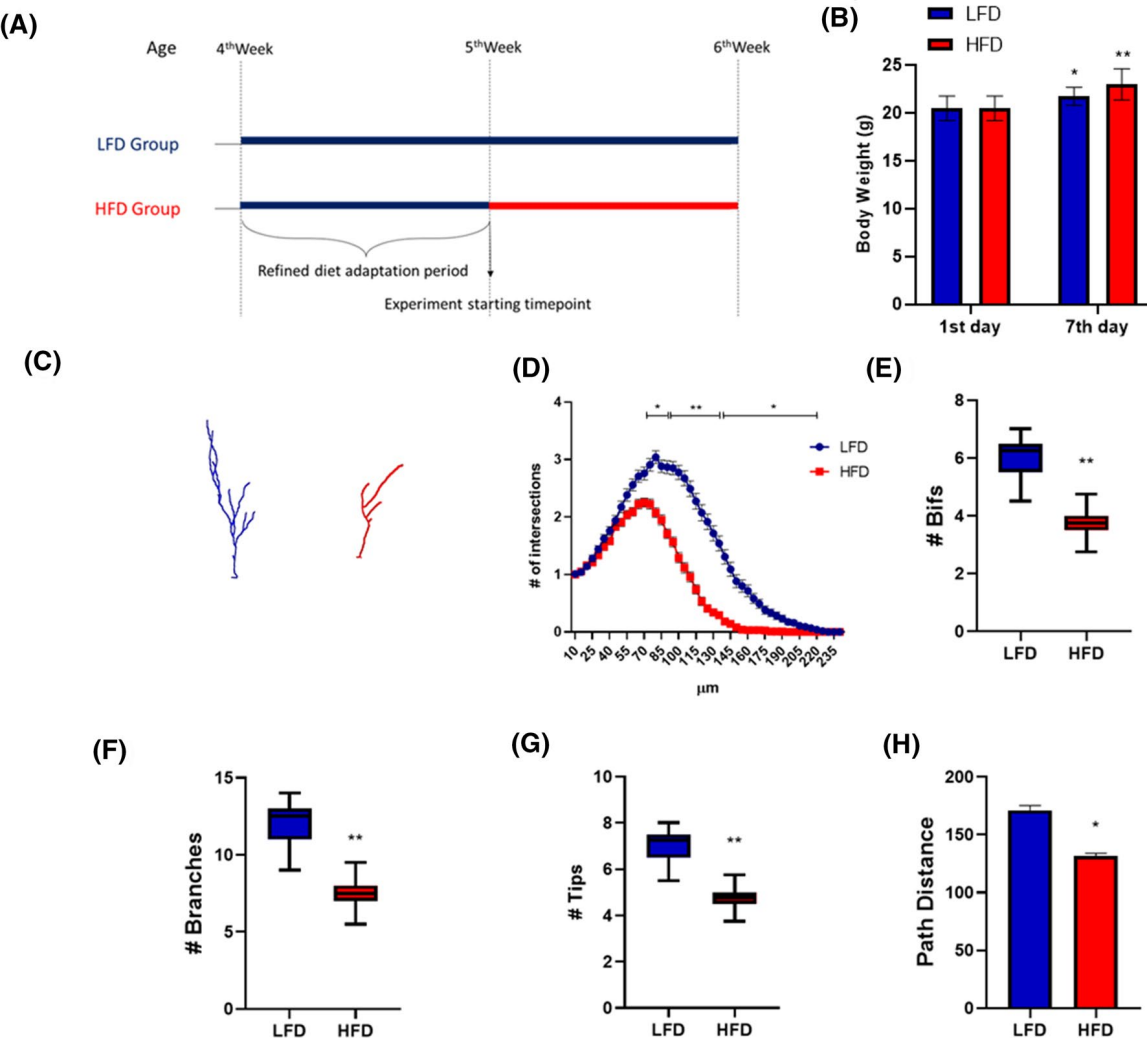
7-day HFD affects cellular complexity of DCX immunolabeled cells in dorsal hippocampus. Schematic experimental design (**A**). Animal body weight at the beginning of the experiment (1st day of diet, 5 weeks of age) and after 7 days of relative diet (**B**). Data are represented as mean ± SEM. *p < 0.05, **p < 0.01 vs. 1st day corresponding group value. Two-way ANOVA with Tukey’s multiple comparisons test. Representative DCX^+^ cell 3D morphological reconstruction in the dorsal hippocampus of animals fed with a LFD (Blue) or HFD (Red) for 1 week (**C**). Sholl intersections profiles of DCX immunolabeled cells of the dentate gyrus of the dorsal hippocampus of animals fed for 7 days with HFD (Red) or LFD (Blue) starting from the fifth week of age (n = 4 animals per group) (**D**). Data are presented as mean ± SEM. *p < 0.05, **p < 0.01 vs. LFD, nested ANOVA on linear mixed-effect model, with animal as a random effect. Summary of morphometric parameters illustrating the total number of bifurcations (**E**), the total number of branches (**F**), the total number of terminal tips (**G**) and the path distance (**H**) of DCX immunolabeled cells of the dentate gyrus of the dorsal hippocampus of animals fed for 7 days with HFD (Red) or LFD (Blue) starting from the fifth week of age (n = 4 animals per group). Data are represented as Box and Wiskers graph (**E–G**) or bar graph (mean ± SEM) (**H**). *p < 0.05, **p < 0.01 vs. LFD, ANOVA on linear mixed-effect model, with animal as a random effect. ImageJ FIJI software (version 1.52) (https://imagej.nih.gov/ij/) was used to produce cell 3D morphological reconstruction. GraphPad Prism 8 (https://www.graphpad.com/) and Power Point were used to generate the figure.

5-week-old C57Bl/6 male mice were exposed to a short period (7 days) of HFD (n = 4) or LFD (n = 4) (Fig. 1A). As shown in Fig. 1B, a statistically significant increase in body weight was observed in both HFD and LFD mice at the end of the diet regimens when compared to day 1 for each experimental group, without differences between the two dietary protocols. Food intake (g/mouse/day) and caloric intake (kcal/mouse/day) were higher in HFD than in LFD group, and data are reported in Table 1.

**Table 1:** Food and Caloric intake of mice fed 7 days with LFD or HFD.

	Food intake (g/mouse/day)	Caloric intake (kcal/mouse/day)
LFD	2.60 ± 0.23	11.11 ± 1.00
HFD	3.09 ± 0.18^#^	17.69 ± 1.02^###^

^#^p < 0.05, ^###^p < 0.001 vs. LFD.

At the end of the diet period, mice were perfused, and their brains removed for immunolocalization studies of the doublecortin (DCX) protein in the dentate gyrus (DG) of the hippocampal formation. In the supra-pyramidal blade of the DG, we selected thirty DCX^+^ immature neurons with their cell body located in the inner third of the granular cell layer (GCL) and a dendritic tree reaching the molecular layer (ML). Sholl analysis allows to resolve on a one-dimensional representation (Sholl Intersection Profile, SIP) the complexity of any given three-dimensional cellular structure (Fig. 1C). For this reason we used it to evaluate the complexity of dendritic arborizations of DCX^+^ cells^23^. We initially evaluated the effects of LFD and HFD on DCX^+^ immature neurons in the dorsal DG (from Bregma − 0.94 to − 2.46 mm). In this region, DCX^+^ cells of HFD animals displayed a remarkable reduction in SIP compared to LFD mice, with a statistical difference observed at 80–220 µm distance from soma (Fig. 1D). This difference was further confirmed by a reduction in additional dendritic morphometric parameters, namely the total number of bifurcations, branches, and tips (Fig. 1E–G, p < 0.01 for all parameters) as well as by a decrease in the length of the longest dendrite referred to as path distance (Fig. 1H, p < 0.05).

Based on these results, in 5-week-old male mice, 7 days of HFD are sufficient to produce a dramatic reduction in the complexity and length of the dendritic tree of DCX^+^ immature neurons in the GCL of dorsal DG.

### HFD effects on the morphological complexity of DCX^+^ immature neurons are transient and reversible

**Figure 2:**
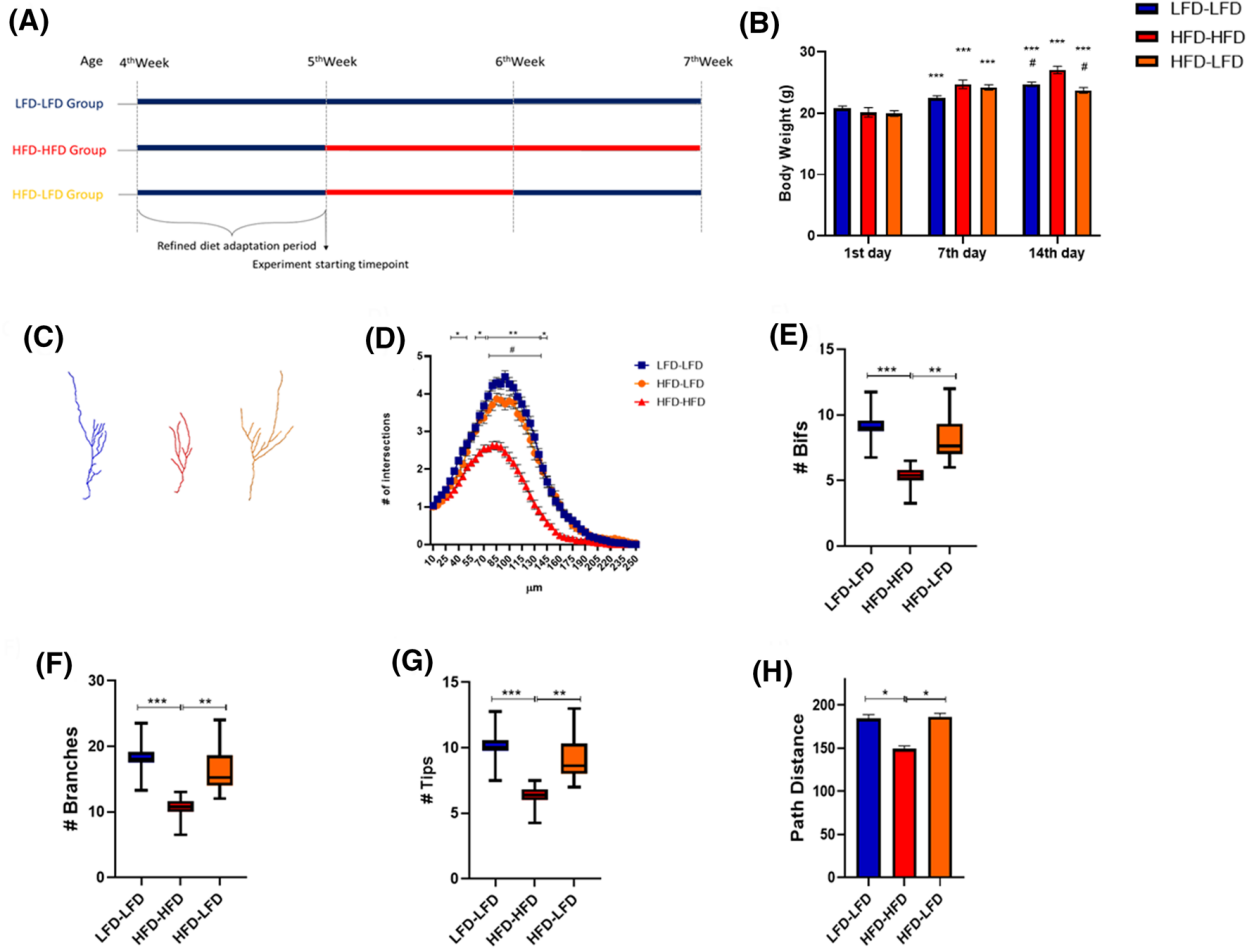
7-day HFD effects on the morphological complexity of DCX^+^ cells are reversible. Experimental design (**A**). Animal body weight at the beginning of the experiment (1st day of diet, 5 weeks of age) and after 7 and 14 days of relative diet (**B**). Data are represented as mean ± SEM. ***p < 0.001 vs. 1st day corresponding group value. ^#^p < 0.05 vs. HFD-HFD at the same timepoint. Two-way ANOVA with Tukey’s multiple comparisons test. Representative DCX^+^ cell 3D morphological reconstruction in the dorsal hippocampus of animals fed with a LFD (Blue) or HFD (Red) for 2 weeks or HFD for 1 week followed by LFD for another week (Orange) (**C**). Sholl intersections profiles of DCX immunolabeled cells of the dentate gyrus of the dorsal hippocampus of animals fed for 14 days with HFD (Red) or LFD (Blue) or for 7 days with HFD followed by 7 days with LFD (Orange) starting from the fifth week of age (n = 4 animals per group) (**D**). Data are presented as mean ± SEM. *p < 0.05, **p < 0.01 LFD-LFD vs. HFD-HFD; ^#^p < 0.05 HFD-LFD vs. HFD-HFD, nested ANOVA on linear mixed-effect model, with animal as a random effect. Summary of morphometric parameters illustrating the total number of bifurcations (**E**), the total number of branches (**F**), the total number of terminal tips (**G**) and the path distance (**H**) of DCX immunolabeled cells of the dentate gyrus of the dorsal hippocampus of animals fed for 14 days with HFD (Red) or LFD (Blue) or for 7 days with HFD followed by 7 days with LFD (Orange) starting from the fifth week of age (n = 4 animals per group). Data are represented as Box and Wiskers graph (**E**–**G**) or bar graph (mean ± SEM) (**H**). *p < 0.05, **p < 0.01, ***p < 0.001, ANOVA on linear mixed-effect model, with animal as a random effect. ImageJ FIJI software (version 1.52) (https://imagej.nih.gov/ij/) was used to produce cell 3D morphological reconstruction. GraphPad Prism 8 (https://www.graphpad.com/) and Power Point were used to generate the figure.

Since a 7 day-period of HFD results in remarkable morphological changes in adult born DCX^+^ cells, we then tested whether this effect may be reversible. A second group of 5-week-old mice (n = 12) were subjected to a 7 day-long HFD supply followed by a return to LFD for 7 days (HFD-LFD group). Animals fed for 14 days with LFD (LFD-LFD group, n = 11) or HFD (HFD-HFD group, n = 11) were used as controls (experimental design summarized in Fig. 2A). As shown in Fig. 2B, we observed a weekly increase in body weight in all diet regimens if compared to day 1. At the end of study (14 days timepoint), a statistical difference in body weight between HFD-HFD group and the animals subjected to other diet regimens was present (LFD-LFD and HFD-LFD, p < 0.05). Food and caloric intake were also calculated in all experimental groups, as reported in Table 2.

**Table 2:** Food and Caloric intake of mice fed 14 days with LFD or HFD or 7 days with HFD followed by 7 days with LFD.

	Food intake (g/mouse/day)		Caloric intake (kcal/mouse/day)	
	1st week	2nd week	1st week	2nd week
LFD-LFD	2.83 ± 0.19	2.99 ± 0.11	12.11 ± 0.83	12.79 ± 0.47
HFD-HFD	3.26 ± 0.32^#^	3.54 ± 0.36^#^	18.68 ± 1.82^###^	19.07 ± 1.02^###^
HFD-LFD	3.20 ± 0.12^#^	2.77 ± 0.10	18.33 ± 0.71^###^	12.63 ± 1.69

^#^p < 0.05, ^###^p < 0.001 vs. LFD-LFD.

After DCX immunostaining of brain sections, morphometric analysis was performed as previously described (n = 4 animals per group) (Fig. 2C). Compared to the LFD-LFD mice, 14 days of HFD led to a marked reduction of the complexity of DCX^+^ immature neurons, with a statistical difference between 65 and 140 µm from soma (Fig. 2D), and to a significant reduction in the total number of bifurcations, branches, tips, and path distance (Fig. 2E–H). Conversely, in the HFD-LFD group we observed no significant difference in cellular complexity compared to LFD-LFD mice (Fig. 2D). Once again, our results were corroborated by L-measure software analysis. Indeed, the total number of bifurcations, branches, tips (Fig. 2E–G) as well as path distance (Fig. 2H) were not different in HFD-LFD and LFD-LFD mice. Altogether these data suggest that the impairment in the dendritic tree complexity of DCX^+^ cells associated with a short period of HFD is a transient event and it can be fully reversed by return to a normo-caloric diet.

**Figure 3:**
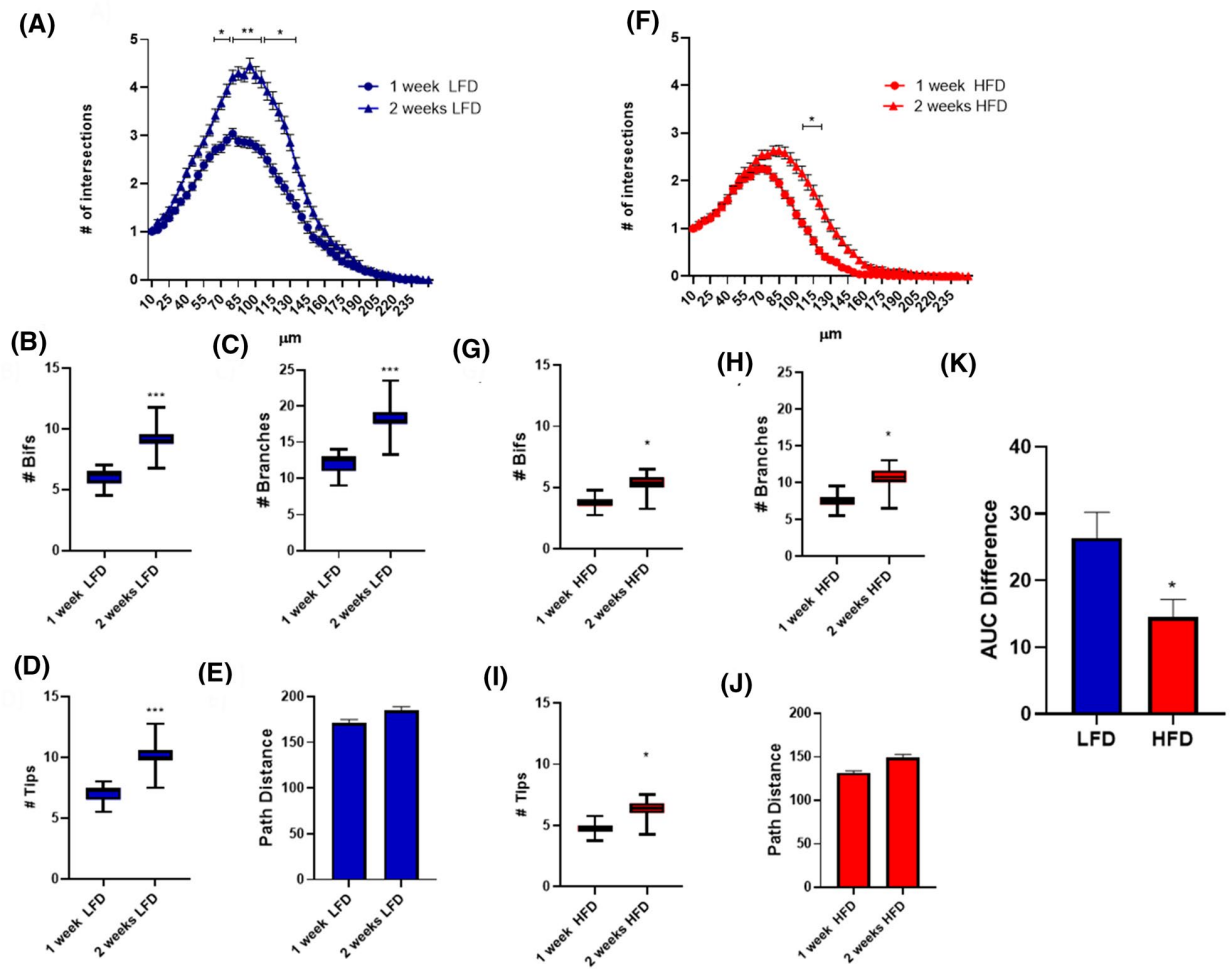
Dendritic complexity of DCX^+^ cells in mice receiving the same dietary regimen for 7 or 14 days. Sholl intersections profiles of DCX immunolabeled cells in the suprapyramidal blade of the dentate gyrus of the dorsal hippocampus of animals fed for 7 or 14 days with LFD (**A**) or HFD (**F**) starting from the fifth week of age (n = 4 animals per group). Data are presented as mean ± SEM. *p < 0.05, **p < 0.01vs. 1 week, nested ANOVA on linear mixed-effect model, with animal as a random effect. Summary of morphometric parameters illustrating the total number of bifurcations (**B**,**G**), the total number of branches (**C**,**H**), the total number of terminal tips (**D**,**I**) and the path distance (**E**,**J**) of DCX immunolabeled cells of the dentate gyrus of the dorsal hippocampus of animals fed for 7 or 14 days with LFD or HFD starting from the fifth week of age (n = 4 animals per group). Data are represented as Box and Wiskers graph (**B**–**D**,**G**–**I**) or bar graph (mean ± SEM) (**E**,**J**). *p < 0.05, ***p < 0.001 vs. 1 week, ANOVA on linear mixed-effect model, with animal as a random effect. Difference in the area under the curve (AUC) of the SIP of DCX^+^ cells in dHP of animals fed for 2 or 1 week with LFD or HFD (**K**). Data are represented as bar graph (mean ± SEM). *p < 0.05 vs. LFD. GraphPad Prism 8 (https://www.graphpad.com/) and Power Point were used to generate the figure.

To complement our analysis, we compared the dendritic complexity of DCX^+^ cells in mice receiving the same dietary regimen for 1 or 2 weeks. Compared to 1 week of diet administration, both LFD and HFD fed animals showed a statistical increase in DCX^+^ cells SIP after 2 weeks of relative diet supply (Fig. 3A,F). Except for path distance (Fig. 3E,J), additional morphometric parameters, i.e. the number of bifurcations, branches and tips (Fig. 3B–D,G–I) confirmed data from Sholl analysis. Although in both LFD- and HFD-fed animals we observed an increase in the SIP after 2 weeks compared to 1 week of diet supply, in HFD mice this increase was significantly reduced compared to LFD mice, as demonstrated by comparing AUC differences (Fig. 3K).

### Short HFD does not affect cellular complexity of DCX^+^ immature neurons in the ventral hippocampus

Our initial analysis was performed in the dorsal DG due to the involvement of this hippocampal subregion in cognitive functions. We then extended our analysis to the complexity of the dendritic tree of immature DCX^+^ cells in the ventral DG (from Bregma − 2.54 to − 4.04 mm).

**Figure 4:**
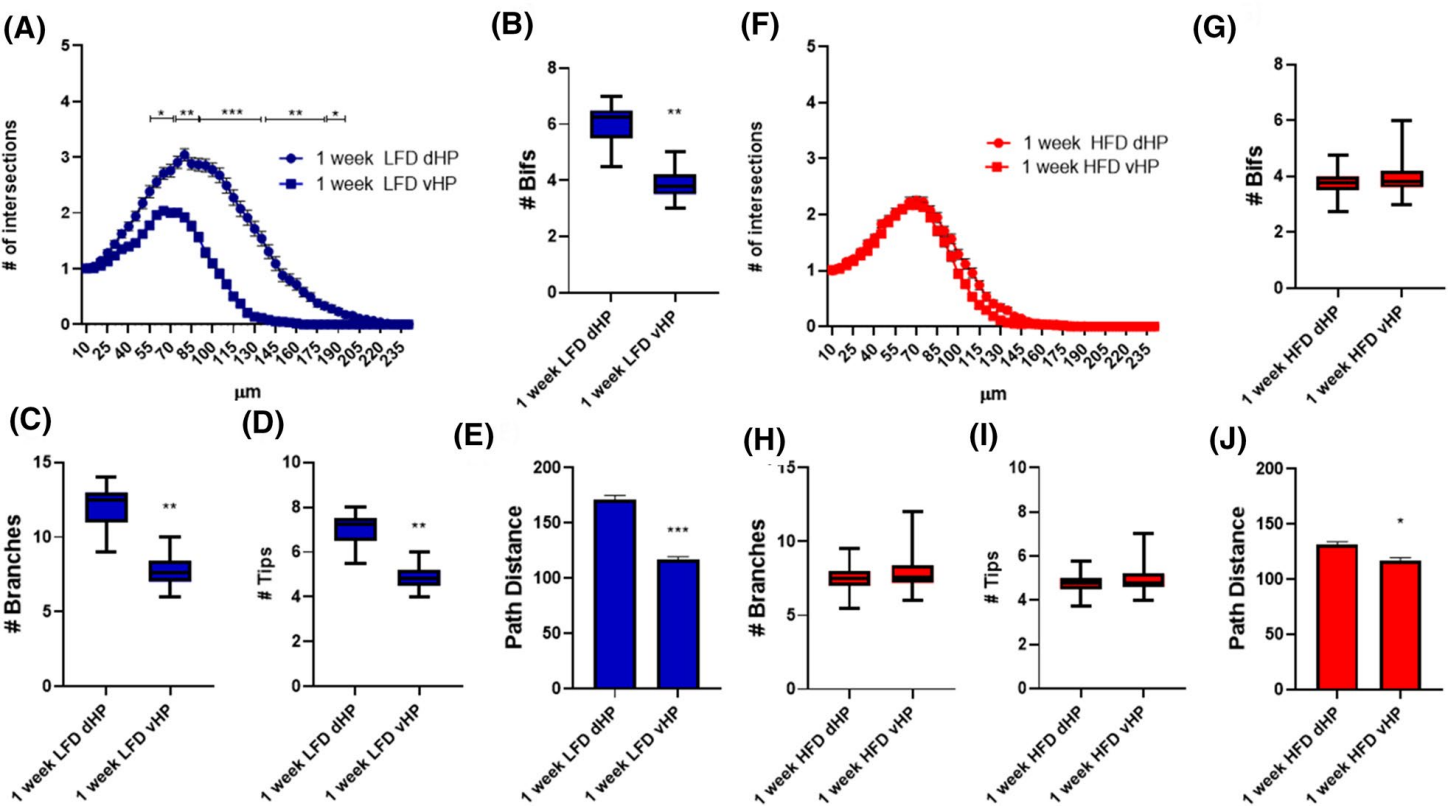
Dendritic complexity of DCX^+^ immature neurons in dorsal and ventral hippocampus of mice receiving the same dietary regimen for 7 days. Sholl intersections profiles of DCX immunolabeled cells in the suprapyramidal blade of the dentate gyrus of the dorsal or ventral hippocampus of animals fed for 7 days with LFD (**A**) or HFD (**F**) starting from the fifth week of age (n = 4 animals per group). Data are presented as mean ± SEM. *p < 0.05, **p < 0.01, ***p < 0.001 vs. dHP, nested ANOVA on linear mixed-effect model, with animal as a random effect. Summary of morphometric parameters illustrating the total number of bifurcations (**B**,**G**), the total number of branches (**C**,**H**), the total number of terminal tips (**D**,**I**) and the path distance (**E**,**J**) of DCX immunolabeled cells of the dentate gyrus of the dorsal or ventral hippocampus of animals fed for 7 days with LFD or HFD starting from the fifth week of age (n = 4 animals per group). Data are represented as Box and Wiskers graph (**B**–**D**,**G**–**I**) or bar graph (mean ± SEM) (**E**,**J**). * p < 0.05, ** p < 0.01 vs. dHP, ANOVA on linear mixed-effect model, with animal as a random effect. GraphPad Prism 8 (https://www.graphpad.com/) and Power Point were used to generate the figure.

Previous studies suggested differences in DCX^+^ cells maturation rate along the dorso-ventral axis of hippocampus^24^. We first analyzed potential differences in dendritic tree complexity of DCX^+^ cells in dorsal and ventral DG of animals fed with either LFD or HFD for 1 week. As described in Fig. 4, in LFD animals DCX^+^ cells displayed a more complex dendritic tree in dorsal hippocampus if compared to the corresponding cell population in the ventral DG, as shown by SIP (Fig. 4A). In addition, a statistically significant increased number of bifurcations, branches, tips (Fig. 4B–D) as well as in path distance (Fig. 4E) was present in dorsal compared to ventral immature neurons. Conversely, in HFD fed mice, differences were observed neither in SIP (Fig. 4F) nor in the number of bifurcations, branches, tips in dorsal vs ventral DCX^+^ cells (Fig. 4G–I). Only path distance was decreased (p < 0.05) in DCX^+^ cells in ventral compared to dorsal DG of HFD mice.

**Figure 5:**
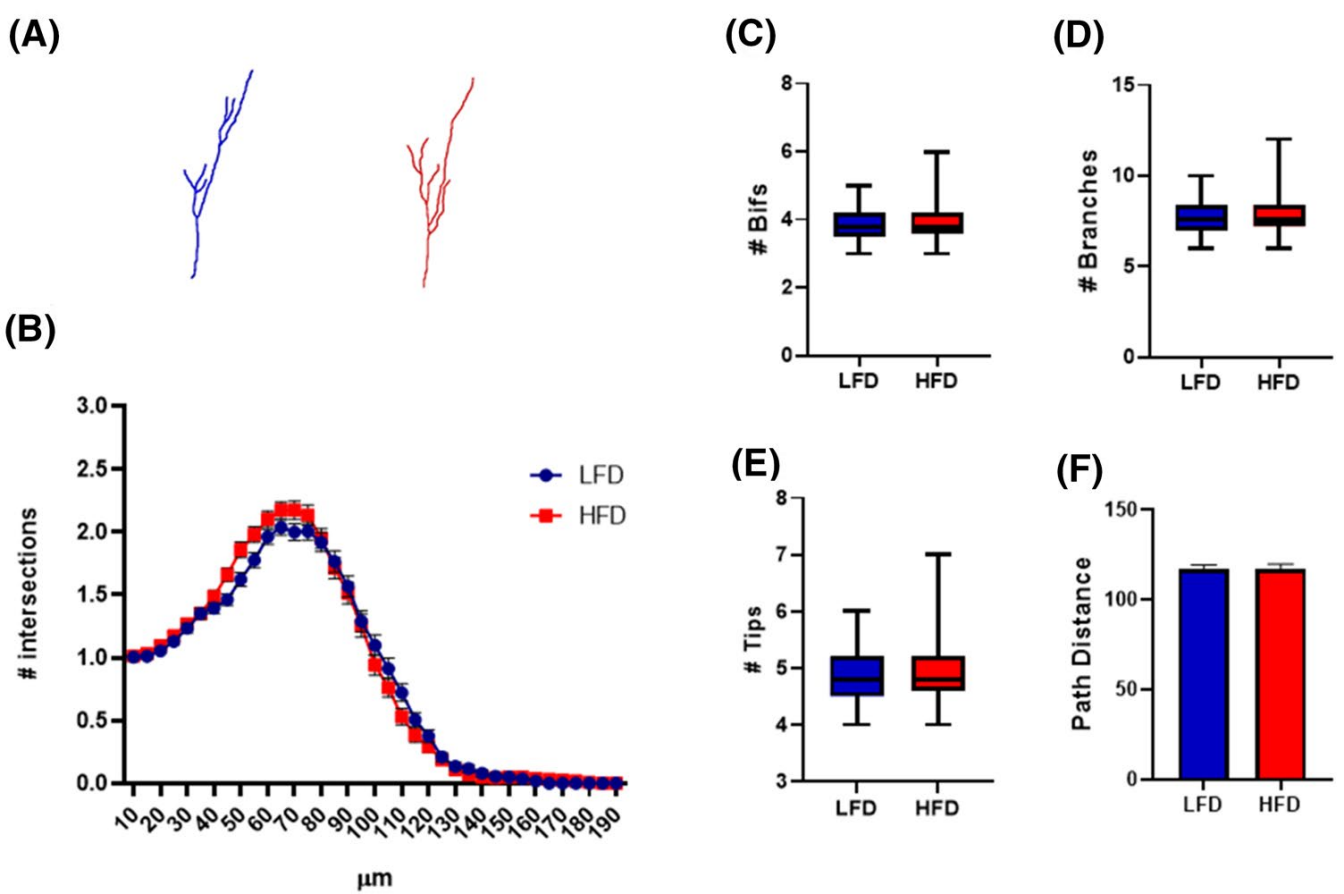
No effect of 7-day HFD on cellular complexity of DCX^+^ cells in the ventral hippocampus. Representative DCX^+^ cell 3D morphological reconstruction in the ventral hippocampus of animals fed with a LFD (Blue) or HFD (Red) for 1 week (**A**). Sholl intersections profiles of DCX immunolabeled cells of the dentate gyrus of the ventral hippocampus of animals fed for 7 days with HFD (Red) or LFD (Blue) starting from the fifth week of age (n = 4 animals per group) (**B**). Data are presented as mean ± SEM. Summary of morphometric parameters illustrating the total number of bifurcations (**C**), the total number of branches (**D**), the total number of terminal tips (**E**) and the maximum reached extension from soma (**F**) of DCX immunolabeled cells of the dentate gyrus of the ventral hippocampus of animals fed for 7 days with HFD (Red) or LFD (Blue) starting from the fifth week of age (n = 4 animals per group). Data are represented as Box and Wiskers graph (**C**–**E**) or bar graph (mean ± SEM) (**F**). ImageJ FIJI software (version 1.52) (https://imagej.nih.gov/ij/) was used to produce cell 3D morphological reconstruction. GraphPad Prism 8 (https://www.graphpad.com/) and Power Point were used to generate the figure.

Finally, we compared morphometric parameters of immature neurons in the ventral DG of animals fed 1 week with LFD or HFD (Fig. 5A). No statistically significant difference was displayed in the two experimental groups, as demonstrated by both Sholl analysis (Fig. 5B) and L-measure software analysis (Fig. 5C–F). These findings suggest that: (I) in juvenile mice dorsal DG immature neurons display more complex and longer dendritic trees than corresponding ventral cells; (II) 7 days HFD reduces complexity of DCX^+^ cells in dorsal and not in ventral DG; (III) HFD mice have DCX^+^ immature neurons with similar complexity in dorsal and ventral DG.

### BDNF expression changes correlate with HFD-associated impairment of DCX^+^ cells in dorsal hippocampus

**Figure 6:**
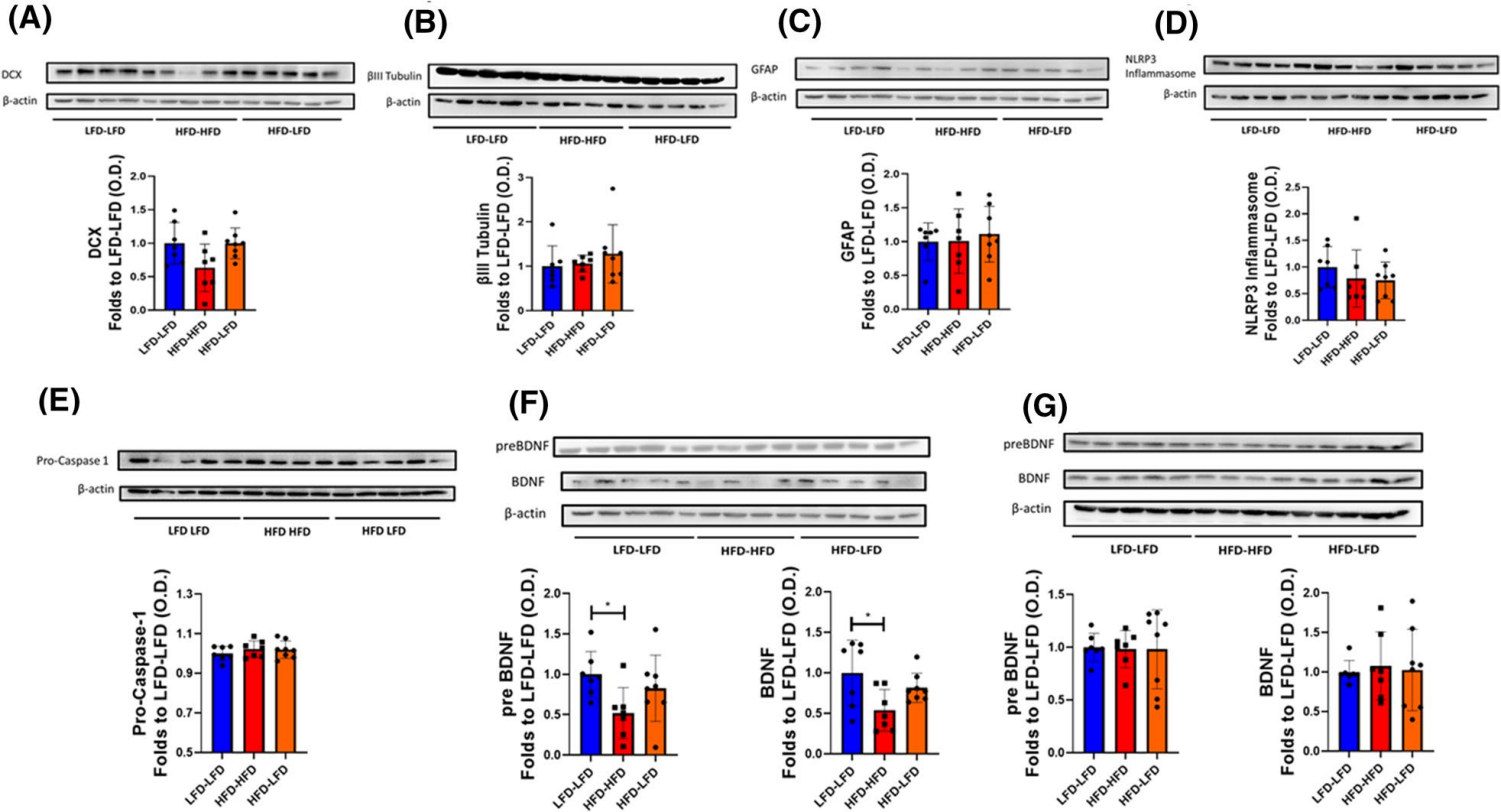
Biochemical changes associated with HFD in dorsal and ventral hippocampus. DCX (**A**), β III Tubulin (**B**), GFAP (**C**), NLRP3 Inflammasome (**D**), Pro-Caspase 1 (**E**) and BDNF (**F**) western blot analysis of the dorsal hippocampus of animals fed for 14 days with HFD (Red) or LFD (Blue) or for 7 days with HFD followed by 7 days with LFD (Orange) starting from the fifth week of age (n = 7 LFD-LFD, n = 7 HFD-HFD, n = 8 HFD-LFD). BDNF (**G**) western blot analysis of the ventral hippocampus of animals fed for 14 days with HFD (Red) or LFD (Blue) or for 7 days with HFD followed by 7 days with LFD (Orange) starting from the fifth week of age (n = 7 LFD-LFD, n = 7 HFD-HFD, n = 8 HFD-LFD). Bands shown are representative. Data are presented as mean ± SD and a single dot represent the result from one animal. *p < 0.05, One-way ANOVA with Tukey’s post-hoc test. Full-length blots are presented in Supplementary Figs. S5–S12. GraphPad Prism 8 (https://www.graphpad.com/) and Power Point were used to generate the figure.

We decided to correlate changes in DCX^+^ cell dendritic tree complexity in response to HFD with alterations in the expression levels of several proteins which are relevant in neuroplasticity of the postnatal and adult hippocampus. Previous studies reported that HFD may impact on the number of DCX^+^ cells^25^ and/or the expression levels of this protein which is often used as a marker of adult born neuroblasts^26–28^. We performed western blot analysis of DCX expression in the dorsal hippocampus of animals subjected to HFD and relative controls. Although there was a trend for reduction in the HFD-HFD experimental group, no statistically significant change in DCX protein levels was observed in dorsal hippocampus of these mice compared to LFD-LFD and HFD-LFD (LFD-LFD vs. HFD-HFD, p = 0.07; HFD-LFD vs. HFD-HFD p = 0.08) (Fig. 6A).

Similarly, we did not observe changes in the expression levels of βIII tubulin, another established marker for newly born neuroblasts^28^ (Fig. 6B).

Since long and short term HFD are associated with neuroinflammation^29–31^ which may result in negative effects in adult born neurons/neuroblasts^32^, we also evaluated if a short period of HFD is sufficient to activate an astrocyte-associated response via changes in glial fibrillary acidic protein (GFAP) expression. As shown in Fig. 6C, neither 14 d-HFD nor 7 d-HFD followed by 7 days of LFD significantly affected GFAP expression in the dorsal hippocampus. Similarly, the NLRP3 inflammasome pathway has been shown to be increased in association with HFD in several tissues including brain^33^. Western blot analysis demonstrated that, in our experimental setting, both NLRP3 and Pro-Caspase 1 protein expression levels were not different in the dorsal hippocampal region of HFD and LFD mice (Fig. 6D,E). Finally, we analyzed the expression of Brain Derived Neurotrophic Factor (BDNF), a signaling molecule which is strongly associated with neuronal trophism and with neuroplasticity/neurogenesis^34^. When the HFD-HFD group was compared to LFD-LFD mice, we observed a statistically significant decrease in the expression of both its active dimeric and precursor forms in the dorsal (Fig. 6F, p < 0.05) but not in the ventral (Fig. 6G) hippocampus. Although in the dorsal hippocampus both pre- and dimeric BDNF protein levels in the HFD-LFD group are higher than in the HFD-HFD group, this difference was not statistically significant (p = 0.20 for the precursor form and p = 0.18 for the active dimeric form HFD-LFD vs HFD-HFD group).

## Discussion

Diet is a modifiable lifestyle factor and a large array of experimental evidence suggests that nutrition might have a direct effect on brain function. Longitudinal studies in humans identified associations between specific nutrients or dietary habits and brain-volume loss^11,12,35^ or brain integrity^36,37^.

Memory is particularly affected by diet in adults^38^. Overweight children and adolescents also show cognitive impairments^39,40^. This observation is of paramount importance since adolescence represents a crucial period for the maturation of brain structures involved in cognition^41^. A large array of preclinical studies in animal models support the link between HFD and impaired learning and memory performance^42–45^.

The hippocampus plays a prominent role in cognition. Evidence that HFD can affect neuron development is provided by many studies that demonstrated a negative impact on hippocampal structure and functions^46^. The dentate gyrus (DG) is also a site for postnatal and adult hippocampal neurogenesis (hNG). A large body of data suggests that, when dysregulated, hNG may contribute to cognitive impairment^16^. Additionally, reduced hNG has been demonstrated in rodents chronically exposed to HFD^25,47–50^. Interestingly, the negative impact of HFD on hNG appears to be influenced by age^47^. Specifically, juvenile age appears particularly vulnerable to HFD. In rodents HFD in this age period has been associated with deteriorated relational and spatial memory performance (as reviewed in Ref.^50^) and reduced hNG^47^.

Formation of newborn hippocampal neurons from neural stem/progenitor cells is a process that takes several weeks to be completed^51^. Previous work demonstrated that long term HFD (2–6 months) in adult rodents results not only in reduced hNG, but in a remarkable reduction in the number of neuroblasts and immature neurons which express the marker doublecortin (DCX) in the dentate gyrus^19,20,47^. The peak of DCX expression in newborn DG cells is usually detected at around 10 days after cell division and decreases at day 30^52^. During their physiological maturation process, which takes weeks, DCX^+^ cells change their orientation, migrate from the subgranular zone to the GCL and their morphological complexity increases, with the dendritic tree growing progressively into the ML of the DG^51,53^. In other words, morphological features within the DCX^+^ cell population in the DG reflect their maturation state. We focused our attention on a DCX^+^ population of immature neurons about 3-week-old according to their soma position within the GCL and dendritic tree reaching the ML. This cell population, whose architectural features has been previously shown to be influenced by environmental stimuli^54^, appeared appropriate for studying the potential effects of short periods of LFD or HFD on their late maturation phase in 5 week-old mice, an age approximately corresponding to adolescence in humans^55^.

Specifically, we analyzed fine structural modifications in the cytoarchitecture of the dendritic tree of such DCX^+^ immature neurons using Sholl analysis and additional morphometric parameters, including length and the number of branches, terminal tips, and bifurcations.

Herein we demonstrated, for the first time, that a 7-day period of HFD is sufficient to negatively affect the dendritic complexity and length of DCX^+^ immature neurons in adolescent male mice. Interestingly, these effects occurred specifically in the dorsal, but not in the ventral DG. Our findings are of relevance since the hippocampus is a complex structure with anatomical and functional segregation along the dorso-ventral axis. The dorsal hippocampus is indeed associated primarily with cognition, while the ventral hippocampus with emotional behavior and response to stress^13,56^.

Also the functional contribution of adult-born neurons varies along the dorsoventral axis. As an example, it has been demonstrated that in a contextual discrimination task, ablation of hNG only in the dorsal, but not ventral, murine DG results in delayed acquisition of discrimination^57^. Conversely, adult‐born neurons in the ventral, but not dorsal, DG are negatively affected by chronic stress^58^. Last but no least several clinically relevant drugs, including antidepressants, specifically promote hNG in ventral hippocampus^15,27,58^. The finding that, in our experimental setting, we observed structural modifications of DCX^+^ cells selectively in the dorsal hippocampus could potentially infer that a short period of high-caloric nutrition may preferentially affect hippocampal- and neurogenesis-dependent functions, including cognition. Region specificity in the impact of HFD was demonstrated also in chronic HFD studies. Four month-long HFD reduced the number of DCX^+^ cells only in the dorsal DG of mice that started diet at 4 weeks of age^22^.

Our study showed a basal difference in immature neurons complexity between dorsal and ventral hippocampus. Cells from the DG of the dorsal hippocampus of animals fed with LFD showed a much greater dendritic complexity than those in the ventral hippocampus. However, despite the strict inclusion criteria described in the materials and methods section and in Supplementary Figs. 2 and 3, we cannot completely rule out that some tips/dendrites of cells included in our analysis might have been cut due to sectioning. This could cause a potential bias especially regarding dorsal vs. ventral hippocampus comparison. The orientation of the dendritic tree of DCX^+^ cells, indeed, could go along with the curve-shaped structure of the hippocampus, but coronal sectioning, although is widely used to study neurogenesis in this area^59^, might not properly follow its curvature along its dorsal–ventral axis. Thereby, sectioning may have differently cut the dendritic tree of immature neurons between dorsal and ventral DG (depending on cell natural orientation). This could represent a possible source of bias. On one hand, it is possible that some non-intact cells might have been accidentally included in our analysis. These cells could appear less complex in the ventral DG (vs. dorsal) not due to their real maturation stage but due to coronal sectioning. On the other hand, we might have restricted our analysis only to those cells with less complex arborization, since more mature DCX^+^ cells may have been cut by coronal sectioning in ventral DG, and thus excluded from our analysis. Nevertheless, differences in morphological complexity of DCX^+^ cells between dorsal vs ventral DG described herein are consistent with other studies that demonstrated a delayed maturation of new born neurons in the ventral compared to dorsal DG in young 9 week-old rats^24^. Moreover, the difference between immature neurons from dorsal and ventral DG was not observed in HFD-fed animals, potentially suggesting that the HFD selectively slowed the development of dorsal DCX^+^ cells.

Herein we also demonstrated that the effects of short HFD on the complexity of DCX^+^ immature neurons in the dorsal hippocampus can be completely reversed by a return to a 1-week LFD. Our in vivo results are in line with recent proteomic studies in 12-week old C57Bl/6J male mice^60^. Specifically, after 3 or 7 days of an HFD regimen, rapid changes in protein expression profile were demonstrated in the hippocampal region. Interestingly, these changes were reversed by a return to a low fat diet^60^. The switch to a normo-caloric diet (3 month-long) was also associated with restored levels of hNG in rats that underwent 3 months of HFD, starting in adolescence^48^.

Interestingly, during the analysis, we also observed that DCX^+^ immature neurons displayed a significantly more complex dendritic tree in mice undergoing 1 week vs. 2 weeks of LFD. One possible interpretation is that 2 weeks of LFD, per se, may promote cellular maturation, compared to 1 week LFD. DCX^+^ cells complexity is also significantly higher after 2 weeks of HFD, compared to 1 week of the same regimen. Interestingly though, the difference in morphological complexity between 2 and 1 week-long HFD is remarkably reduced compared to the difference between 2 weeks and 1 week LFD. Altogether, we believe that this observation supports the idea that short periods of HFD affect maturation of immature DCX^+^ neurons differently compared to corresponding periods of LFD. Our data, indeed, potentially suggest that, unlike LFD, short HFD may counteract and/or slow down the late physiological maturation of DCX^+^ immature neurons.

A limitation of our study is that all mice were switched from chow to low fat diet 1 week prior the beginning of the experimental protocol, so to allow animals to adapt to a refined diet and ensure comparable basal conditions among LFD/HFD groups, for example in terms of food texture which may slightly differ between chow and refined diets. It is possible that 1 week of diet adaptation, switching from a chow (hard-diet) to a low-fat diet (softer-diet) may impact DCX^+^ cells complexity as this modification is taking place approximately 1 week before they reach the immature stage, during their differentiation process. Since a chow group was not included in the experimental design for direct comparison with HFD and LFD groups, we cannot exclude the possibility that reduction in complexity of DCX^+^ immature neurons in the HFD group is caused by interaction of food texture and high fat content rather than by diet alone.

In our study we attempted to correlate structural changes in the dorsal DCX population with biochemical events triggered by HFD in a region-specific manner. We analyzed expression levels of DCX and also βIII tubulin, another marker of immature neurons, and we did not observe any significant changes in these proteins in the dorsal hippocampus of HFD- versus LFD-diet treated mice. This result strengthens the relevance of morphometric studies like ours to appreciate fine structural changes in newborn cells that are undergoing maturation.

Chronic HFD has been associated with changes in glia number, morphology and/or function in several brain regions^61,62^. In our experimental setting, no changes were also observed in the expression levels of the astrocytic marker GFAP, NLRP3 inflammasome and Pro-caspase 1 in dorsal hippocampus of HFD mice. However, due to the very short time of diet supply, in our experimental conditions we cannot rule out that changes in the mRNA for these inflammatory markers may have occurred in absence of protein changes.

Several molecular mechanisms, both extrinsic and intrinsic, allow for temporal and spatial control of dendritic arborizations of new born neurons^63^. Among molecules participating in the modulation of dendritic architecture, brain derived neurotrophic factor (BDNF), is a relevant one. BDNF is a key neurotrophin with important hippocampal-dependent functions, including cognition^64^. Additionally, the positive modulatory role of BDNF in hNG and in the cytoarchitecture of newborn neurons is also a very consolidated concept (reviewed in Ref.^65^). In rodents HFD long-term chronic intake, reduces BDNF content in many brain areas, including the hippocampus, while nutritional restriction increases brain BDNF content^66–68^.

Herein we demonstrated that HFD was associated with a significant reduction in the expression of the active and the precursor forms of BDNF specifically in the dorsal and not in the ventral hippocampus of adolescent mice, if compared to animals fed a LFD. Interestingly, BDNF changes in dorsal hippocampus were partially reverted by a return of HFD-treated mice to 1 week of LFD (even if the difference between HFD-HFD and HFD-LFD group do not reach a statistical difference). These findings suggest that the reduction of BDNF levels may potentially contribute to HFD-associated reduction in the complexity, length and decrease of the number of branches, tips and bifurcations of the dendritic tree of DCX^+^ cells.

Previous studies suggest that BDNF influences maturation of new hippocampal cells. High levels of BDNF are expressed in rodent hippocampi and dendritic development of newborn granule neurons is disrupted in BDNF conditional mutants^69^. Within the DG, it has been shown that BDNF receptor TrkB is expressed by neural stem cells and DCX^+^ cells and that in the latter cells its expression is closely linked to progression toward neuronal maturation^70^. Based on these observations, BDNF may directly and positively influence physiological maturation of DCX^+^ immature neurons and its deficiency, that we associated to short HFD in dorsal hippocampus, may contribute to reduce and/or slowdown such maturation in a region-specific manner. Of course, we cannot exclude that also indirect effects, mediated by TrkB expressed on other DG cells, both glial and neuronal, may play a role in diet-associated changes in the DCX population. As far as the cellular source of BDNF, at this stage we can only speculate on this aspect. BDNF is produced and released by neuronal, glial (both astrocytes and microglia), and even endothelial cells. It is quite intriguing that also mature granule cells, which actively release BDNF depending on their activity, can influence maturation of newly born neurons^71^. Therefore, HFD may also affect locally and neuronally-derived BDNF in the dorsal DG. Future studies involving BDNF signal activation in HFD-treated mice should further investigate the contribution of impaired BDNF/TrkB axis, taking advantage of validated pharmacological tools^72^.

At this stage of knowledge, we have no information on intracellular pathways involved in the morphological changes in the DCX^+^ populations of the dorsal DG, including pathways influencing organization of cellular cytoskeleton. Interestingly, in a recent proteomic study, the expression of several cytoskeletal proteins was reduced by 3 or 7 day-HFD in the hippocampus, and particularly proteins which have roles in microtubule stability including microtubule-associated protein 2 (MAP2) and stathmin^60^.

Future studies should be conducted to evaluate whether the fine and reversible morphological alterations elicited by a short HFD in the DCX population also correlate with hippocampal-dependent cognitive performance. It has been demonstrated that short (7–9 days) HFD impaired long-term memory of object location as well as the induction of in vivo LTP in the CA1 in juvenile mice. Interestingly, in the same study, 7-day HFD exposure enhanced in vivo LTP and object location memory in adult rats^73^. Although our current study focused specifically on adolescent mice, in the future we will also evaluate if short HFD differently affects the DCX^+^ population along the dorso-ventral axis of hippocampus at different ages. Furthermore, as a strategy to achieve a more accurate measure of potential differences in dorsal vs ventral evaluation, future analyses will be performed using a sectioning method that combines coronal sectioning, for the dorsal hippocampus, with horizontal sectioning, more suitable for ventral hippocampus (as proposed by Egeland et al.^59^). This could help to follow the natural orientation of cells along the dorsal–ventral axis and contribute to cope with potential sectioning bias.

One of the limitations of our study is the fact that we have analyzed only adolescent male mice. Very recent studies, indeed, found that chronic administration of a HFD (4 months starting from 2 months of age) to mice produces striking sex differences on hNG, and these differences are not likely due to sex differences in diet metabolism^74^. In particular, the study found that HFD significantly reduced cell proliferation and the number of young/immature neurons in female, and not in male mice. In the future we will investigate if short HFD supply will determinate consequences on hippocampal hNG differently not only at distinct ages but also as a function of animal sex.

## Materials and methods

### Animals

Adult male C57BL/6J mice of 4 weeks of age were utilized in two different studies. Mice, kept 3–5/cage with access to water and food ad libitum*,* were housed in a light- (12 h light, 12 h dark) and temperature- (22–24 °C) controlled room in high-efficiency particulate air (HEPA)-filtered Thoren units (Thoren Caging Systems) at the University of Piemonte Orientale animal facility. Animal care and handling were performed in accordance with the Italian law on animal care (D.L. 26/2014), as well as European Directive (2010/63/UE) and ARRIVE guidelines, and approved by the Organismo Preposto al Benessere Animale (OPBA) of University of Piemonte Orientale, Novara, Italy (DB064.61).

### Diet administration protocol

A timeline representation of the experimental design can be found in Figs. 1A and 2A. The composition of LFD and HFD is described in Supplementary Fig. S4.

Study 1: eight animals started receiving a LFD (13% kcal from fat, 67% kcal from carbohydrates, 20% kcal from proteins, Laboratori Piccioni), instead of the normal chow diet provided by the animal facility, starting from the fourth week of age so to get used to a refined diet^75,76^. After 1 week (5 weeks of age), animals were randomly divided into 2 groups: the first group continued to be fed with LFD (LFD group, n = 4), while the second group of animals was fed with a HFD (60% kcal from fat, 21% kcal from carbohydrates, 19% kcal from proteins, Laboratori Piccioni) (HFD group, n = 4). Both groups received LFD or HFD for 1 week.

Study 2: Thirty-four 4-week-old mice underwent LFD as previously described. After 1-week of LFD, animals were randomly divided into 3 groups: the first group continued to be fed with LFD, while the second and third groups of animals were fed with a HFD. After another week, only the third group of animals reverted to a LFD for another week (HFD-LFD group, n = 12), while the first (LFD-LFD, n = 11) and the second (HFD-HFD, n = 11) groups continued with their diet regiment (LFD and HFD respectively).

In both studies all experimental groups switched from chow diet to LFD 1 week prior the beginning of the experimental protocol, so to ensure comparable basal conditions. The use of a refined diet (LFD) for the control groups, and not of chow diet, is required not only for better matching of fiber and nutrient composition, but also of texture between LFD and HFD^77^.

In both studies, animal body weight, and mean values of food/caloric intake per mouse per day were recorded weekly. Briefly, to evaluate food and caloric intake, food was weighted at the beginning of the week and after 7 days (at the same time). The overall consumed food/cage was calculated by subtracting food weight after 1 week from the initial amount of provided diet. This value was divided by the number of days (7) and by the number of mice per cage to obtain a mean of food intake.

### Tissue preparation for IHC analysis

At the end of diet regimen, 8 animals of study 1 and 12 mice from study 2 (n = 4 per each experimental group) were deeply anesthetized with a mix of Zoletil (Zolazepam, 60 mg/kg) and Xylazine (20 mg/kg) i.p. and transcardially perfused with saline and then with paraformaldehyde (PFA) 4% in 0.1 M phosphate buffer pH 7.4, as previously described^78^. After PFA-perfusion, brains were rapidly removed, post-fixed in 4% PFA for 24 h, dehydrated in 15% sucrose for 24 h, and then transferred in sucrose 30% for at least 24 h. Then, 40 μm-thick coronal sections were cut with cryostat and collected in cryoprotectant solution at − 20 °C until use.

### Tissue preparation for western blot analysis

At the end of diet regimen, the remaining animals from study 2 (LFD-LFD group n = 7, HFD-HFD group n = 7, HFD-LFD group n = 8) were euthanized by cervical dislocation and brains were rapidly removed and dissected using a mouse brain matrix (designed for the slicing of coronal sections through the brain at intervals of 1 mm) in order to isolate separately dorsal and ventral hippocampus. Once isolated, dorsal and ventral hippocampi were snap frozen and stocked at − 80 °C until use.

### Immunohistochemistry and image acquisition

Dorsal and ventral hippocampus areas included in the analysis were delineated according to the Paxinos Mouse Brain Atlas^79^ (Supplementary Fig. S1). From a complete series of one-in eight brain sections throughout the dentate gyrus, four corresponding sections for each mouse were selected from Bregma − 0.94 to − 2.46 mm (dorsal hippocampus) or from Bregma − 2.54 to − 4.04 mm (ventral hippocampus). Staining was performed on free floating sections as previously described^80^. Endogenous peroxidase activity was blocked with 0.3% H_2_O_2_ in 0.1 M TBS for 10 min. Sections were subsequently treated at 4 °C for 1 h in a blocking solution containing 10% goat serum (GS) 0.3% Triton X-100 in 0.1 M TBS, pH 7.4, and incubated with guinea pig anti-DCX antibody (1:15,000, Millipore, Ab2253) in 5% GS, 0.1% Triton X-100 in 0.1 M TBS, for 36 h at 4 °C. Then, sections were incubated with biotinylated goat anti-G.Pig secondary antibody (1:150, Vector Laboratories, BA-7000) in 5% GS in 0.1 M TBS for 2 h at 4 °C. Labelled cells were visualized using the ABC system (Vectastain Elite, Vector Laboratories, PK-6100) with 3,3′-diaminobenzidine as chromogen (Sigma-Aldrich, D3939). Images were acquired using a LSM700 laser-scanning confocal microscope (Carl Zeiss, Le Pecq, France), with 20× magnification (objective: EC Plan-Neofluar 20×/0.5 M27) with an image matrix of 1024 × 1024 pixel, a pixel scaling of 0.313 × 0.313 μm and a depth of 8 bit. Confocal-images were collected in Z-stacks with a slice-distance of 0.4 μm.

### 3‑D DCX^+^ cell selection, reconstruction and quantitative morphometry

The image stacks were imported into ImageJ FIJI software (version 1.52), where 3D reconstructions were performed by an investigator blinded to animal groups using the Simple Neurite Tracer plugin^81^ which allows semi-automatic and unbiased reconstruction of cell arborizations, as previously described^27,82–85^. DCX^+^ immature neurons to be reconstructed were selected based on the following criteria: (i) located in the suprapyramidal blade of DG, (ii) with their dendritic tree growing perpendicular to GCL, (iii) with soma located in the inner third of GCL and (iv) with dendrites entering the molecular layer. Cells whose dendrites/tips were detected in the first or the last scan plan of z-stack were excluded from analysis because their tree may be cut during sectioning. Examples of cells that, based on the abovementioned criteria, were included or excluded from the analysis are shown in Supplementary Figs. S2 and S3 respectively. A short Supplementary Video showing frames moving throughout the z-stack of a DCX^+^ cell reconstruction is also provided (Supplementary Video).

30–40 cells per animal (corresponding to 120–160 cells per experimental group), in dorsal and ventral hippocampus were chosen. Morphological analysis was performed on a 3D reconstruction of the DCX^+^ cell with the Sholl analysis plugin^86^, using default settings (enclosing radius cutoff = 1 intersection, Sholl method = linear) with radii increasing by 5 μm. The Sholl Intersection Profile (SIP) counts the number of intersections between cell dendrites and concentric spheres emanating from the center of cell soma. Moreover, 3D reconstructions were exported as SWC files and analyzed with L-measure tool to evaluate additional morphometric features^87^.

### Western blot analysis

Briefly, dorsal and ventral hippocampi were homogenized in RIPA buffer (Tris 50 mM, NaCl 100 mM, EGTA 5 mM, Nonidet NP-40 1%, Dithiothreitol 5 mM, Protease inhibitor cocktail [P8340, Sigma Aldrich] 10 µl/ml, PMSF 1 mM, NaF 25 mM, NaVO_4_ 1 mM) in ice and incubated at 4 °C for 2 h under constant agitation. Samples were then centrifuged at 20,000*g* for 30 min at 4 °C and supernatants were collected. Protein concentration was evaluated with Bradford assay. Forty micrograms of proteins for each sample were separated onto 8% acrylamide gel and then transferred to nitrocellulose membrane. After blocking (2 h in 5% not fat dry milk in TBS 1X), membranes were incubated with rabbit anti-DCX (1:1000, Cell Signaling, 4604), rabbit anti-βIII tubulin (1:5000, Abcam, ab18207), rabbit anti-BDNF (1:2000, Abcam, ab108319), Rabbit anti-NLRP3 inflammasome (1:1000, Cell Signaling, 15101), mouse anti-Caspase 1 (1:1000, Adipogen, AG-20B-0044) and mouse anti-GFAP (1:1000, Millipore, MAB3402) overnight at 4 °C and then incubated with corresponding HRP conjugated secondary antibody (R&D systems, 1:10,000) for 1 h at room temperature. Proteins were detected with an ECL detection system (SuperSignal West Pico PLUS Chemiluminescent Substrate, Thermo) and quantified by densitometry using analytic software (ImageLab software, Biorad). Results were normalized with respect to β-actin densitometric value.

### Statistical analysis

All statistical analysis and data visualizations were performed in GraphPad Prism 8. For statistical analysis of body weight and food and caloric intake, Two-way ANOVA with Tukey’s post-hoc test was used. For statistical analysis of morphological parameters, a linear mixed effects model was used to model the data of each parameter, with animal as a random effect. Using this approach, it is possible to overcome the dependency of the repeated observations within each animal^27^. The presence of significant differences was tested using Nested t-test or one-way ANOVA. SIPs were analyzed by mixed-effects nested ANOVA approach with individual animal as random effect. Western blots results were analyzed with one-way ANOVA with Tukey’s post-hoc test. For all analysis, significance was defined as p < 0.05.

## Supplementary Information

Supplementary Information.

Supplementary Video 1.

## Data Availability

All data are available upon request.
