# Supplementary material for: Short high fat diet triggers reversible and region specific effects in DCX+ hippocampal immature neurons of adolescent male mice
Source: Sci Rep. 2021 Nov 2;11:21499. doi: 10.1038/s41598-021-01059-y (PMC8563989; doi:10.1038/s41598-021-01059-y)
Supplement: Supplementary file 1 — Supplementary Information. [file 41598_2021_1059_MOESM1_ESM.pdf]

Short High Fat Diet triggers reversible and region specific effects

in DCX^+ hippocampal immature neurons of adolescent male mice

Fausto Chiazza [1,2], Heather Bondi [1,2], Irene Masante [1,2], Federico Ugazio [1,2], Valeria Bortolotto [1,2], Pier Luigi Canonico [2], Mariagrazia Grilli [1,2*]

[1] Laboratory of Neuroplasticity, [2] Department of Pharmaceutical Sciences, University of Piemonte Orientale, Novara, Italy

Supplementary Information

CORRESPONDING AUTHOR:

Mariagrazia Grilli, Laboratory of Neuroplasticity, Department of Pharmaceutical Sciences, University of Piemonte Orientale, Novara, Italy, mariagrazia.grilli@uniupo.it

Supplementary Fig. S1.

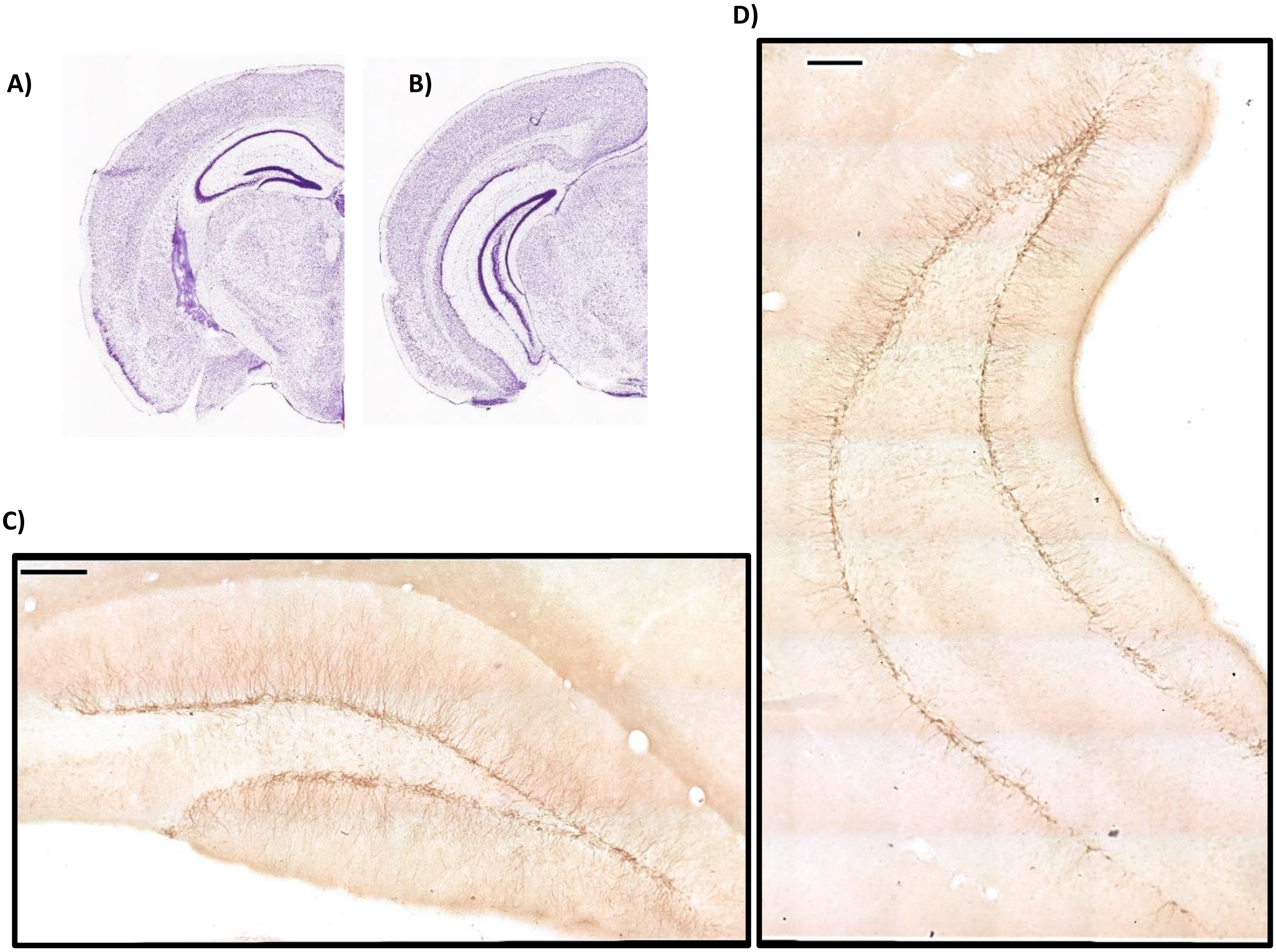

Supplementary Fig. S1: Representative coronal sections containing dorsal (A) and ventral (B) hippocampus (Image credit: Allen Institute, atlas.brain-map.org). Dorsal (C) and ventral (D) dentate gyrus stained for DCX (Scale Bar: 150µm). ImageJ FIJI software (version 1.52) (https://imagej.nih.gov/ij/) was used to produce images. Microsoft PowerPoint was used to generate the figure.

Supplementary Fig. S2

A)

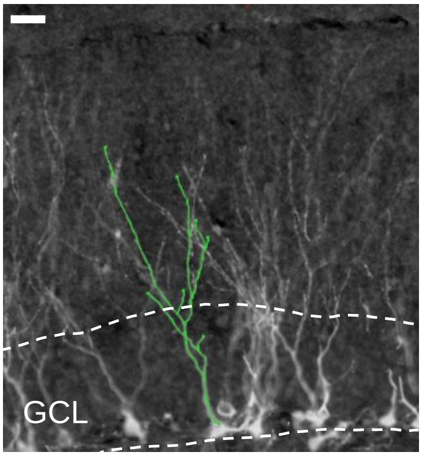

Supplementary Fig. S2: A) Z-projection of the 3D reconstruction (in green) of a DCX⁺ cell included in our analysis. White dotted lines indicate boundaries of granule cell layer (GCL). Scale bar= 20 µm. (B) Confocal images of the z-stack (plans 01-16) for the reconstructed DCX⁺ cell in Supplementary Fig. S2A. For each plan, a region of the dendritic tree (white box) is also shown at higher magnification in a grayscale image (a), with corresponding cell reconstruction (b); scale bar = 10 µm. The “Simple neurite tracer” plugin traced cell ramifications only in 03-13 plans of z-stack, i.e., where cell arborization signal is in-focus. The absence of reconstructed dendrites/tips in the first or last z-stack plans indicates that cell dendritic tree is not cut due to sectioning and, since intact, included for analysis.

ImageJ FIJI software (version 1.52) (https://imagej.nih.gov/ij/) was used to produce images. Microsoft PowerPoint was used to generate the figure.

B)

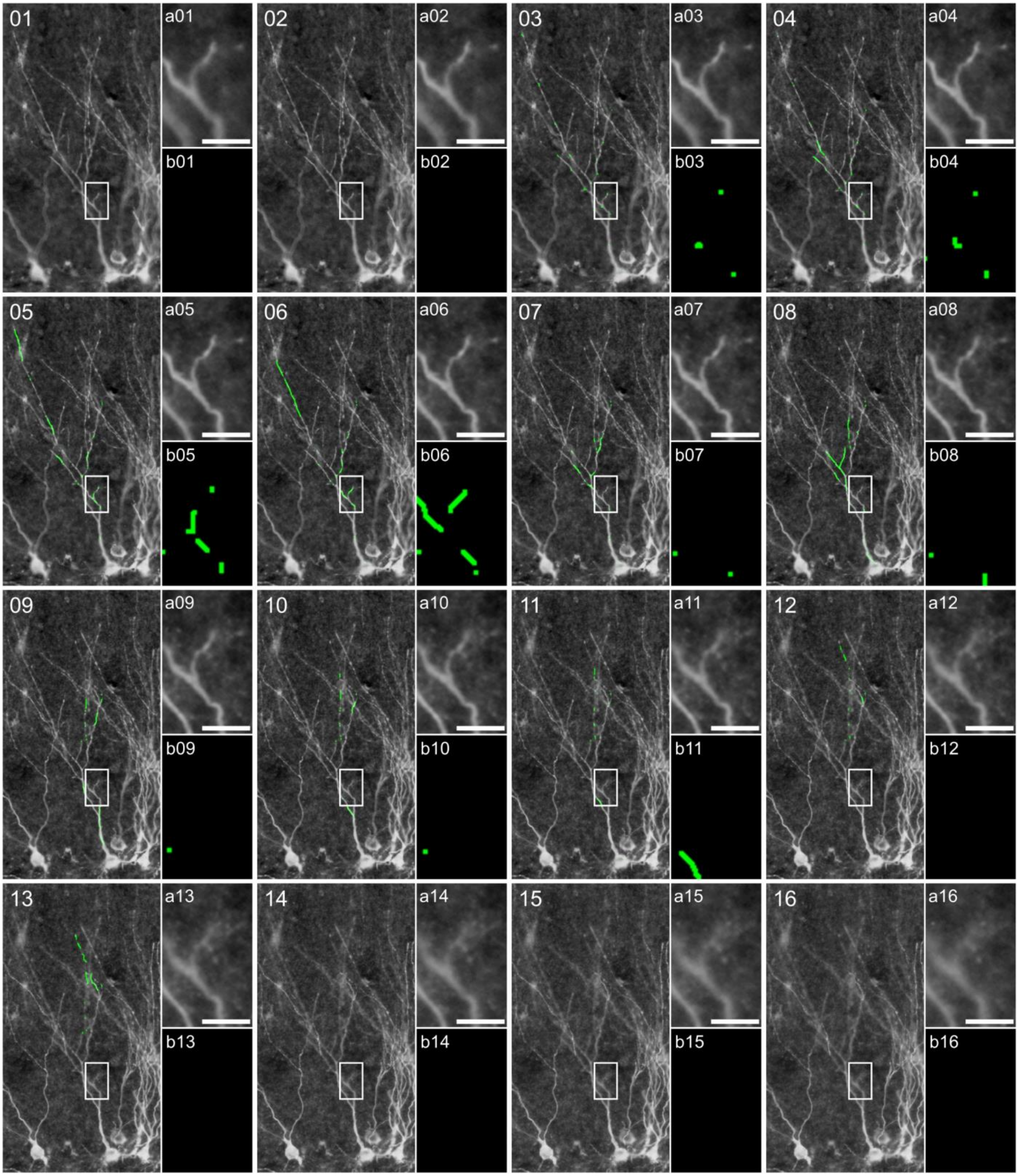

Supplementary Fig. S3

A)

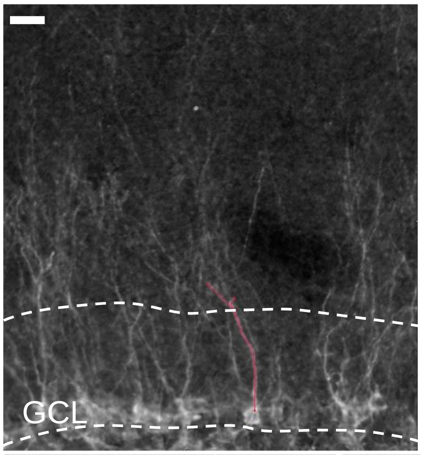

Supplementary Fig. S3: (A) Z-projection of the 3D reconstruction (in red) of a DCX$^+$ cell excluded from our analysis. White dotted lines indicate boundaries of granule cell layer (GCL). Scale bar = 20 µm. (B) Confocal images of the z-stack (plans 01-16) of the reconstructed DCX$^+$ cell in Supplementary Fig. S3A. For each plan, a region of the dendritic tree (white box) is also shown at higher magnification in a grayscale image (a) with corresponding cell reconstruction (b); scale bar = 10 µm. The "Simple neurite tracer" plugin traced cell ramifications in 01-07 plans of z-stack, i.e., where cell arborization signal is in-focus. The presence of reconstructed dendrites/tips in the first plan of the z-stack indicates that the cell dendritic tree is cut due to sectioning and, since not intact, excluded from our analysis.

ImageJ FIJI software (version 1.52) (https://imagej.nih.gov/ij/) was used to produce images. Microsoft PowerPoint was used to generate the figure.

B)

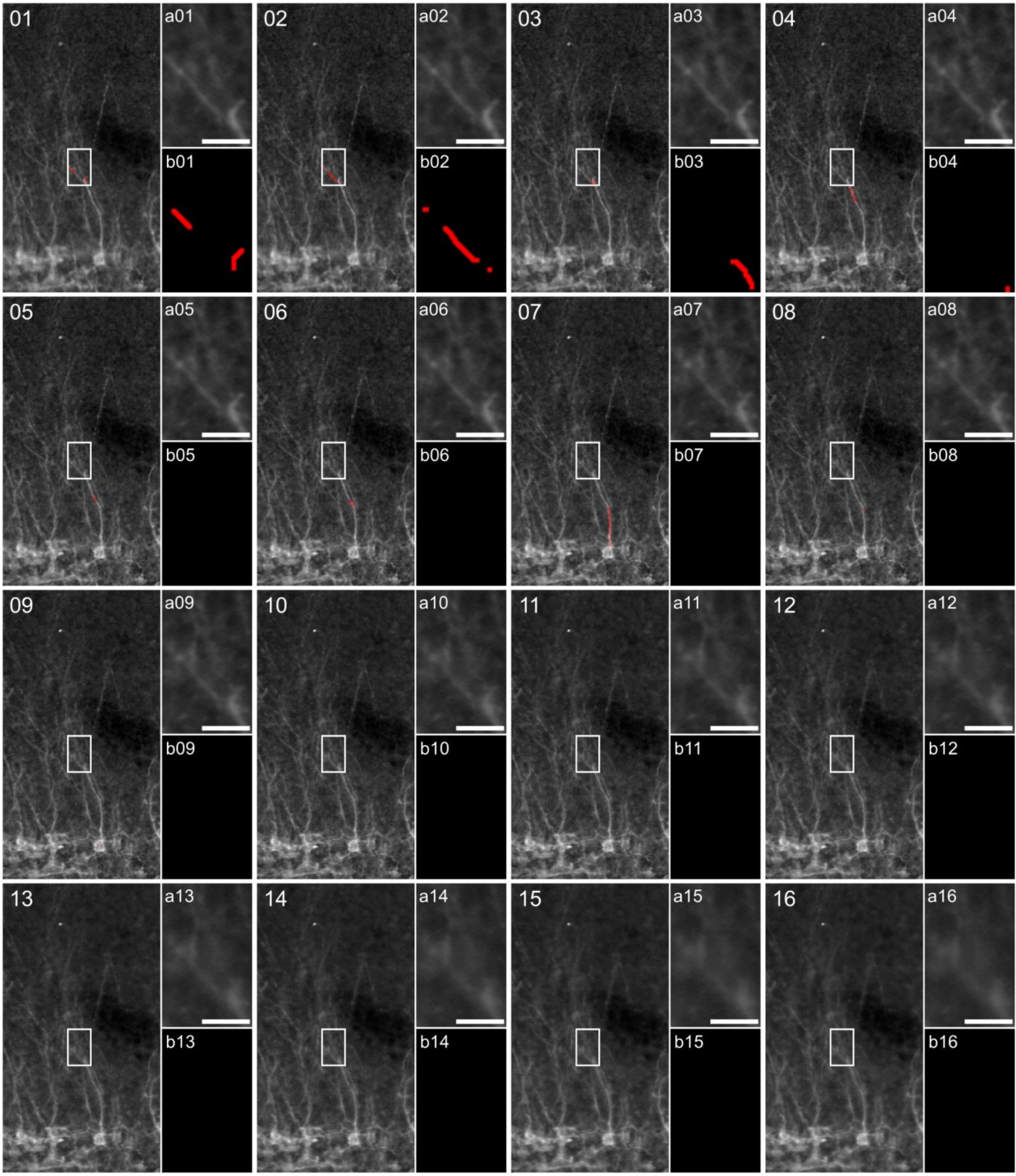

Supplementary Fig. S4

A)

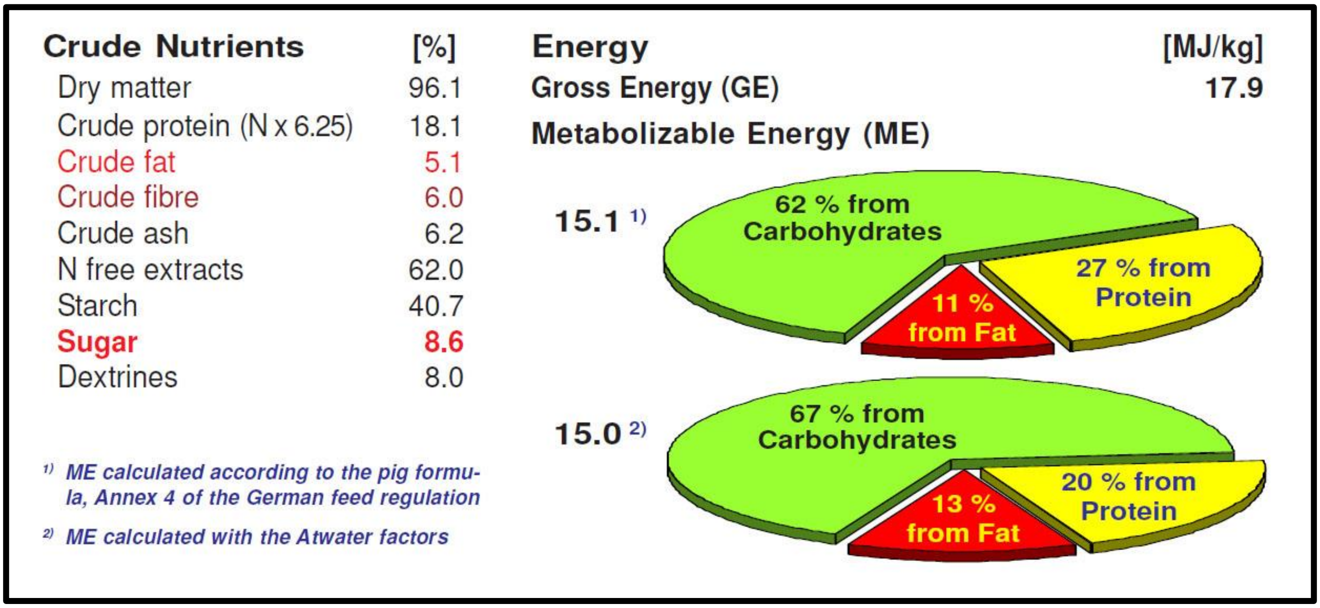

B)

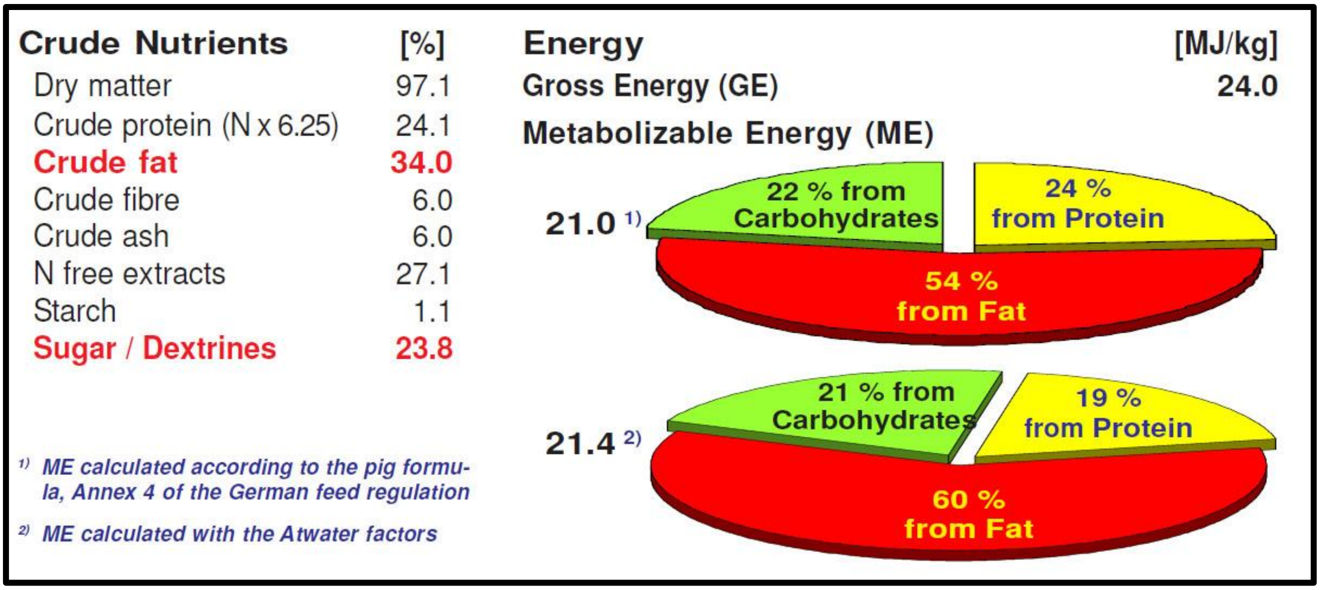

Supplementary Fig. S4: Composition of LFD (A) and HFD (B) (Laboratori Piccioni, https://totofood.it/). Microsoft PowerPoint was used to generate the figure.

Supplementary Fig. S5

DCX (45 kDa)

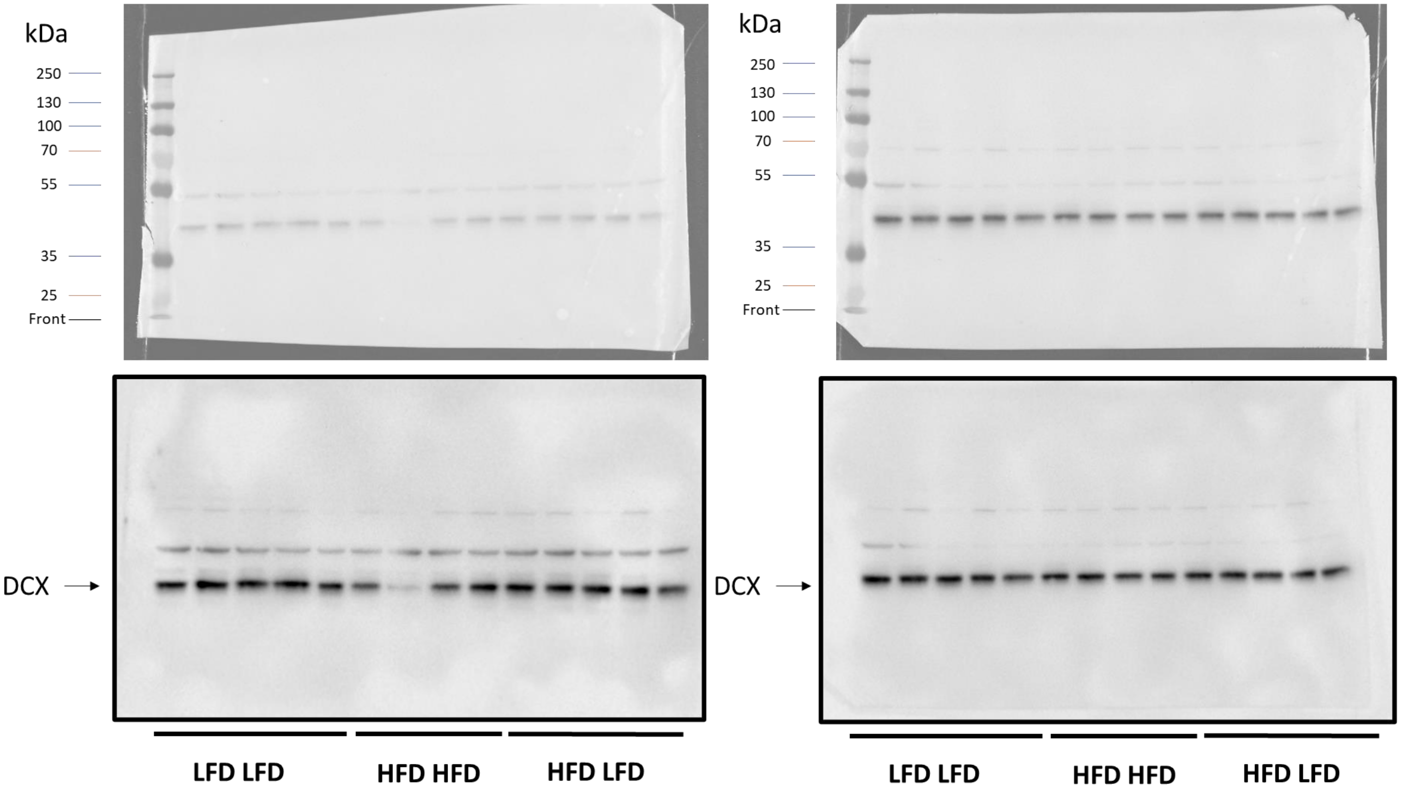

Supplementary Fig. S5: Original whole DCX western blot shown in Fig. 6.
Upper panels: representation of the chemiluminescence bands merged with the colorimetric picture of the WB membrane.
Lower panels: chemiluminescence images with indication of the analyzed bands.
For the final figure in the manuscript, photoshop was used to crop the horizontal line of bands. The whole horizontal lines of bands were adjusted at the same time and placed in Fig. 6. The samples of the 2 blots derive from the same experiment and blots were processed in parallel. Microsoft PowerPoint was used to generate the figure.

Supplementary Fig. S6

Beta III Tubulin (55 kDa)

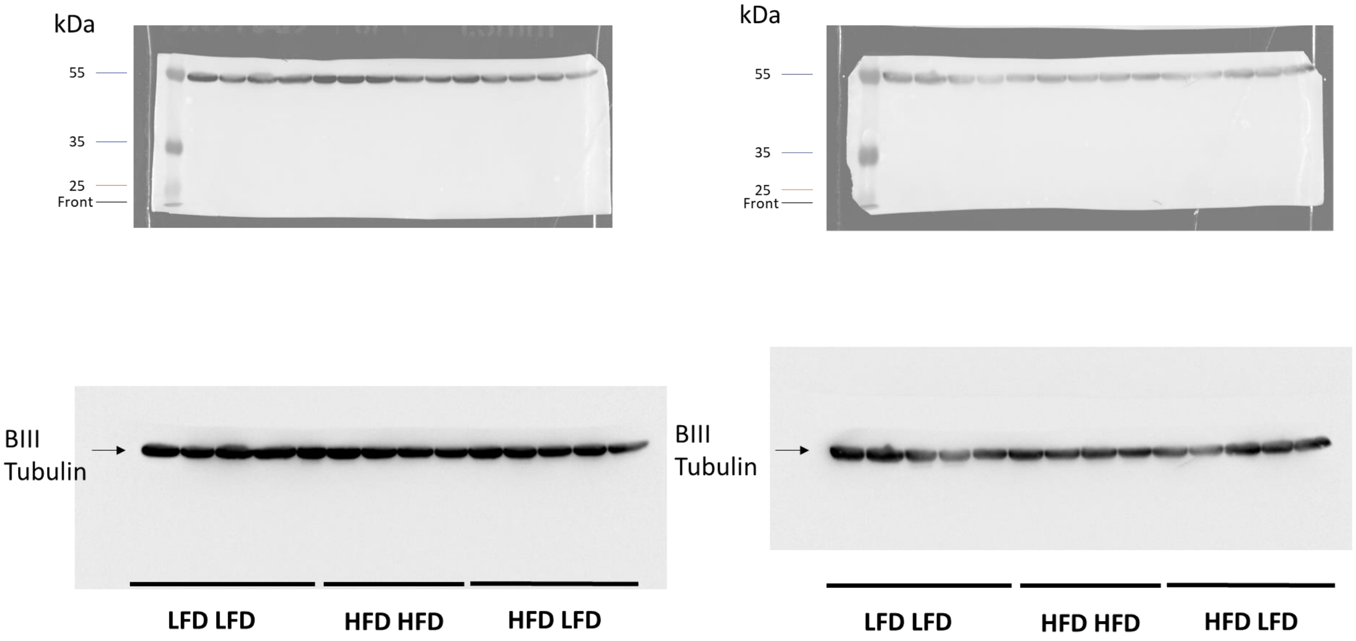

Supplementary Fig. S6: Original whole Beta III Tubulin western blot shown in Fig. 6.
Upper panels: representation of the chemiluminescence bands merged with the colorimetric picture of the WB membrane.
Lower panels: chemiluminescence images with indication of the analyzed bands.
For the final figure in the manuscript, photoshop was used to crop the horizontal line of bands. The whole horizontal lines of bands were adjusted at the same time and placed in Fig. 6. The samples of the 2 blots derive from the same experiment and blots were processed in parallel. Microsoft PowerPoint was used to generate the figure.

Supplementary Fig. S7

GFAP (50 kDa)

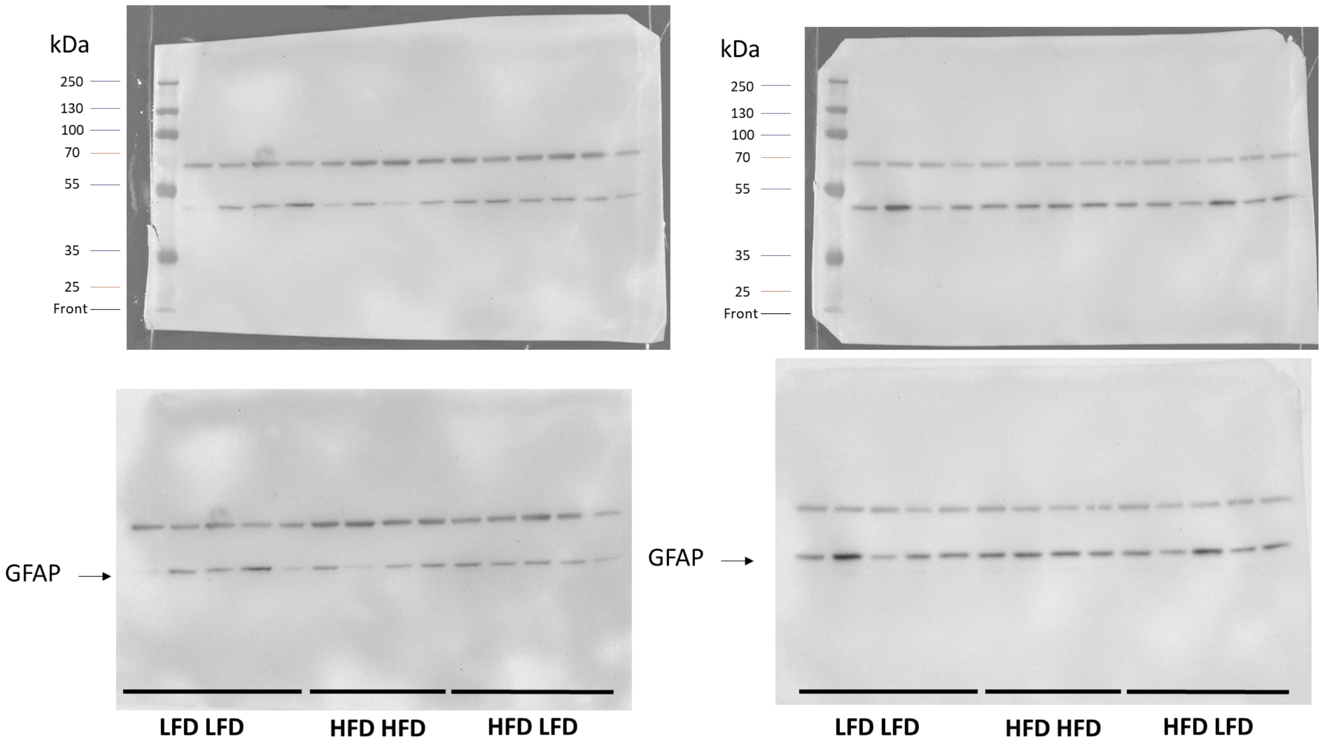

Supplementary Fig. S7: Original whole GFAP western blot shown in Fig. 6.
Upper panels: representation of the chemiluminescence bands merged with the colorimetric picture of the WB membrane.
Lower panels: chemiluminescence images with indication of the analyzed bands.
For the final figure in the manuscript, photoshop was used to crop the horizontal line of bands. The whole horizontal lines of bands were adjusted at the same time and placed in Fig. 6. The samples of the 2 blots derive from the same experiment and blots were processed in parallel. Microsoft PowerPoint was used to generate the figure.

Supplementary Fig. S8

NLRP3 inflammasome (85 kDa)

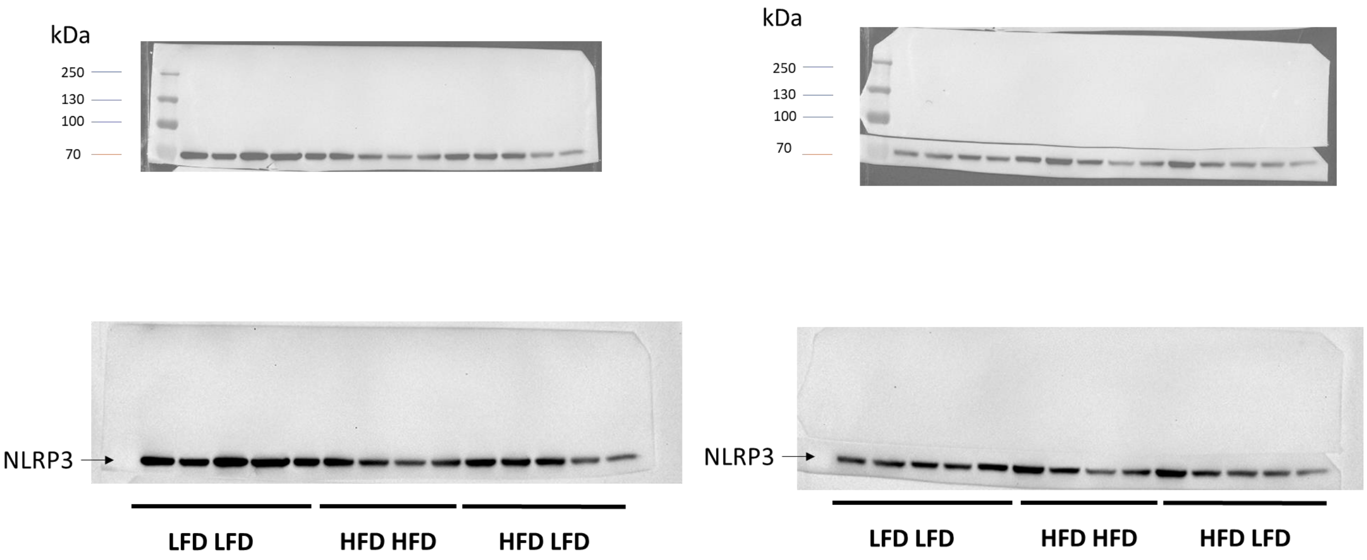

Supplementary Fig. S8: Original whole NLRP3 Inflammasome western blot shown in Fig. 6.
Upper panels: representation of the chemiluminescence bands merged with the colorimetric picture of the WB membrane.
Lower panels: chemiluminescence images with indication of the analyzed bands.
For the final figure in the manuscript, photoshop was used to crop the horizontal line of bands. The whole horizontal lines of bands were adjusted at the same time and placed in Fig. 6. The samples of the 2 blots derive from the same experiment and blots were processed in parallel. Microsoft PowerPoint was used to generate the figure.

Supplementary Fig. S9

Pro-caspase 1 (45 kDa)

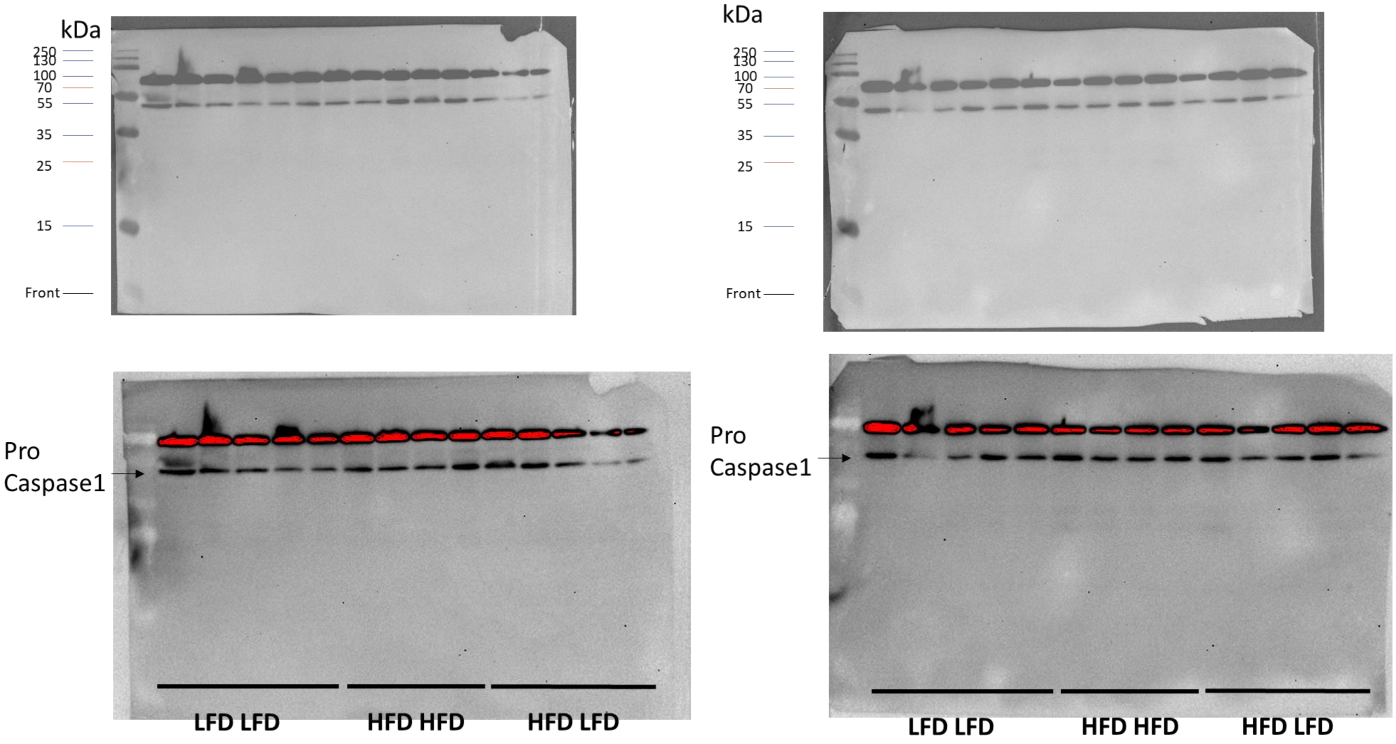

Supplementary Fig. S9: Original whole Pro-Caspase 1 western blot shown in Fig. 6.
Upper panels: representation of the chemiluminescence bands merged with the colorimetric picture of the WB membrane.
Lower panels: chemiluminescence images with indication of the analyzed bands.
For the final figure in the manuscript, photoshop was used to crop the horizontal line of bands. The whole horizontal lines of bands were adjusted at the same time and placed in Fig. 6. The samples of the 2 blots derive from the same experiment and blots were processed in parallel. Microsoft PowerPoint was used to generate the figure.

Supplementary Fig. S10

Pre BDNF dHP (45 kDa)

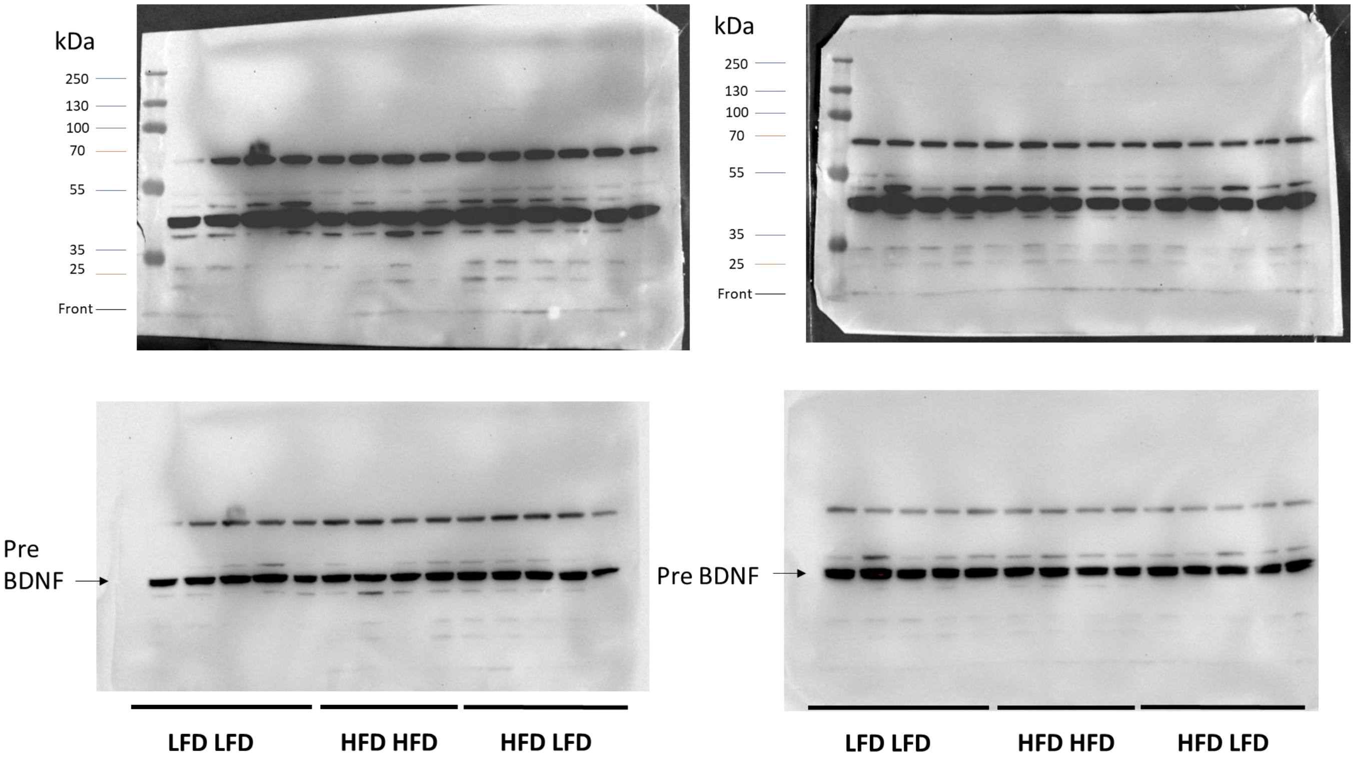

Supplementary Fig. S10: Original whole pre BDNF western blot shown in Fig. 6 (dHP).
Upper panels: representation of the chemiluminescence bands merged with the colorimetric picture of the WB membrane.
Lower panels: chemiluminescence images with indication of the analyzed bands.
For the final figure in the manuscript, photoshop was used to crop the horizontal line of bands. The whole horizontal lines of bands were adjusted at the same time and placed in Fig. 6. The samples of the 2 blots derive from the same experiment and blots were processed in parallel. Microsoft PowerPoint was used to generate the figure.

Supplementary Fig. S11

dimeric BDNF dHP (28 kDa)

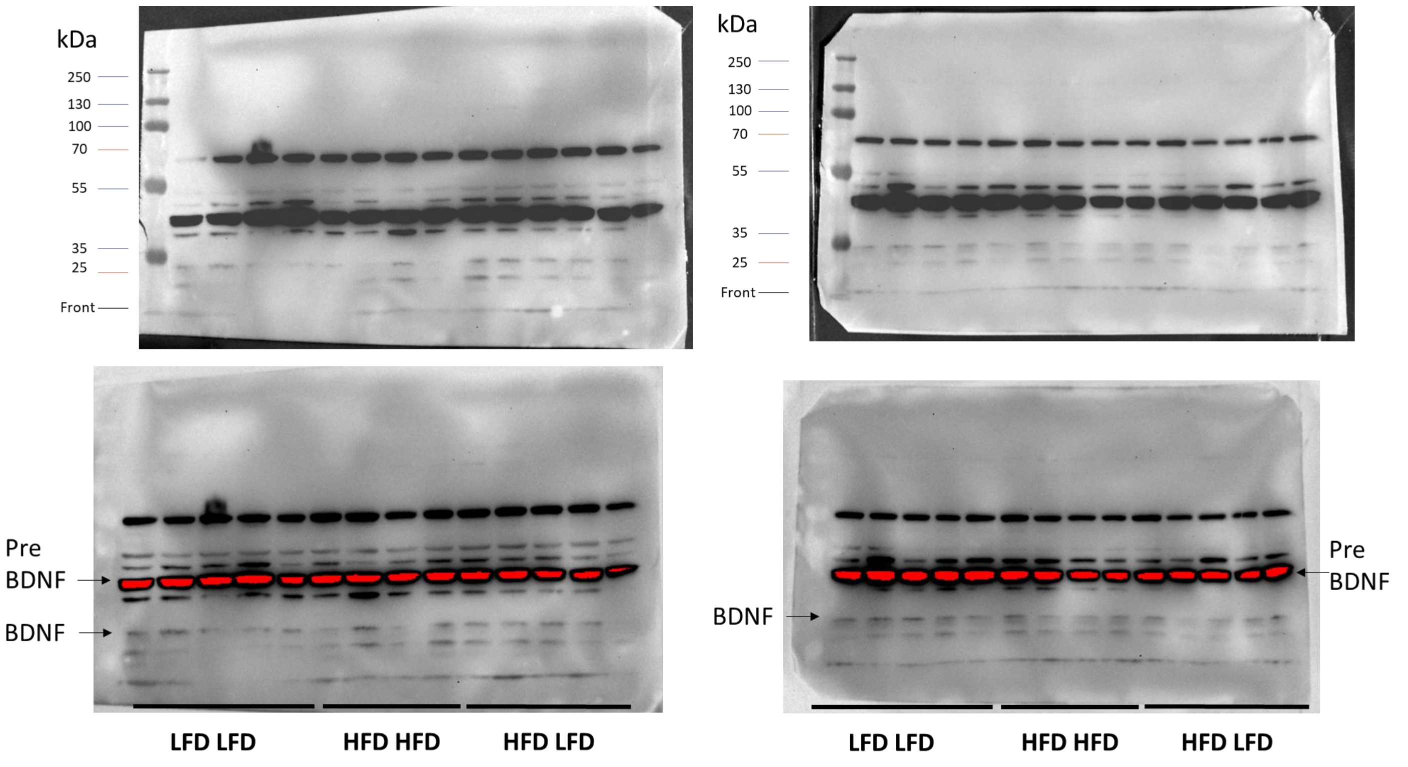

Supplementary Fig. S11: Original whole BDNF western blot shown in Fig. 6 (dHP).
Upper panels: representation of the chemiluminescence bands merged with the colorimetric picture of the WB membrane.
Lower panels: chemiluminescence images with indication of the analyzed bands.
For the final figure in the manuscript, photoshop was used to crop the horizontal line of bands. The whole horizontal lines of bands were adjusted at the same time and placed in Fig. 6. The samples of the 2 blots derive from the same experiment and blots were processed in parallel. Microsoft PowerPoint was used to generate the figure.

Supplementary Fig. S12

Pre BDNF (45 kDa) and dimeric BDNF (28 kDa) vHP

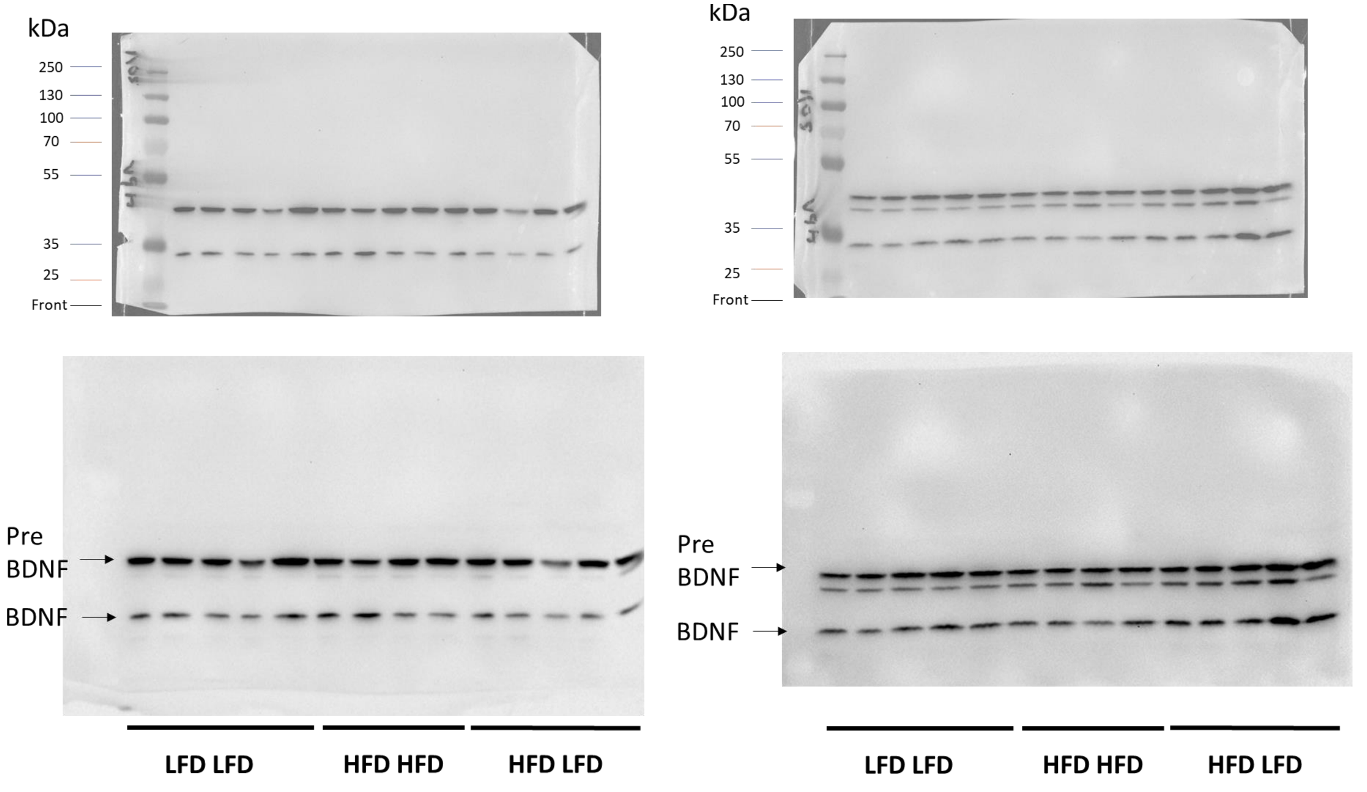

Supplementary Fig. S12: Original whole pre BDNF and BDNF western blot shown in Fig. 6 (vHP).
Upper panels: representation of the chemiluminescence bands merged with the colorimetric picture of the WB membrane.
Lower panels: chemiluminescence images with indication of the analyzed bands.
For the final figure in the manuscript, photoshop was used to crop the horizontal line of bands. The whole horizontal lines of bands were adjusted at the same time and placed in Fig. 6. The samples of the 2 blots derive from the same experiment and blots were processed in parallel. Microsoft PowerPoint was used to generate the figure.

Supplementary Video

Supplementary Video: on the left, confocal grayscale plans (01-16) of the DCX^+ immature neuron of Supplementary Fig. S2A (yellow arrow) moving through the z-stack; on the right, in grey is shown z-projection of cell reconstruction. When arborization signal get in-focus (on the left), corresponding reconstruction is highlighted in green (on the right). Scale bar = 20 µm.

ImageJ FIJI software (version 1.52) (https://imagej.nih.gov/ij/) was used to produce images. Microsoft PowerPoint was used to generate the video.
